# Interrogating DNA methylation associated with Lewy body pathology in a cross brain-region and multi-cohort study

**DOI:** 10.1038/s41467-026-77009-x

**Published:** 2026-09-15

**Authors:** Joshua Harvey, Jennifer Imm, Morteza Kouhsar, Adam R. Smith, Byron Creese, Rebecca G. Smith, Gregory Wheildon, Valentin Laroche, Szi Kay Leung, Leonidas Chouliaras, Gemma Shireby, Zane Jaunmuktane, Eduardo De Pablo-Fernández, Thomas Warner, Debra J. Lett, Djordje Gveric, Hannah Brooks, Johannes Attems, Alan Thomas, Wilma DJ Van de Berg, Emma Dempster, Clive Ballard, John T O’Brien, Dag Aarsland, Jonathan Mill, Lasse Pihlstrøm, Ehsan Pishva, Katie Lunnon

**Affiliations:** 1 Department of Clinical and Biomedical Science, Faculty of Health and Life Sciences, University of Exeter, Exeter, UK; 2 Department of Life Sciences, College of Health, Medicine and Life Sciences, Brunel University of London, London, UK; 3 Department of Psychiatry and Neuropsychology, School for Mental Health and Neuroscience (MHeNS), Maastricht University, Maastricht, The Netherlands; 4 Department of Psychiatry, University of Cambridge School of Clinical Medicine, Cambridge, UK; 5 Specialty Dementia and Frailty Service, Essex Partnership University NHS Foundation Trust, St Margaret’s Hospital, Epping, UK; 6 Great Ormond Street Hospital, University College London, London, UK; 7 Queen Square Brain Bank for Neurological Disorders, University College London Queen Square Institute of Neurology, University College London, London, UK; 8 Newcastle Brain Tissue Resource, Newcastle University, Newcastle, UK; 9 MS and Parkinson’s Tissue Bank, Department of Brain Sciences, Imperial College London, London, UK; 10 The Oxford Brain Bank, University of Oxford, Oxford, UK; 11 Department of Anatomy and Neurosciences, Section Clinical Neuroanatomy and Biobanking, Amsterdam UMC, Vrije Universiteit Amsterdam, Amsterdam, The Netherlands; 12 Amsterdam Neuroscience, Program Neurodegeneration, Amsterdam UMC, Vrije Universiteit Amsterdam, Amsterdam, The Netherlands; 13 Institute of Psychiatry, Psychology & Neuroscience (IoPPN), Kings College London, London, UK; 14 Institute of Clinical Medicine, University of Oslo, Oslo, Norway; 15 Department of Neurology, Oslo University Hospital, Oslo, Norway

**Keywords:** Epigenetics in the nervous system, Parkinson's disease, DNA methylation

## Abstract

Here, we conduct a large meta-analysis of DNA methylation across multiple cortical brain regions, in relation to increasing burden of Lewy body pathology. Utilizing a dataset of 1205 samples (832 unique donors), we identify 24 false discovery rate significant differentially methylated loci associated with Lewy body pathology, with loci located in *UBASH3B* and *PTAFR*, and an intergenic locus passing the genome-wide significance threshold. Ontological enrichment analysis highlights several neurologically relevant traits, including synaptic alterations. We compare methylation signatures between neurodegenerative pathologies and highlight a shared epigenetic profile across Lewy body, Alzheimer’s and Huntington’s diseases, although the top-ranked loci show disease specificity. Utilizing summary statistics from large-scale genome-wide association studies, we identify significant enrichment of DNA methylation differences with respect to increasing Lewy body pathology in the *SNCA* genomic region. We also identify specific relationships between genetic risk variants for Lewy body dementia and Parkinson’s disease to methylation quantitative trait loci in the *SNCA* 5’ region and expression of alternative transcript isoforms.

## Abstract (web summary)

Lewy body pathology is associated with widespread molecular changes in the brain. Here, the authors identify DNA methylation alterations linked to increasing Lewy body burden, revealing disease-specific and shared epigenetic signatures across neurodegenerative disorders and implicating regulatory variation at the SNCA locus.

## Introduction

The Lewy body diseases including Parkinson’s disease (PD) and Dementia with Lewy bodies (DLB), are neurodegenerative diseases classified by the accumulation of alpha-synuclein (α-synuclein) in neurons, forming Lewy bodies (LBs). Collectively, these diseases are the second most common cause of neurodegeneration and dementia, following Alzheimer’s disease (AD) and confer a high care giver burden, given the complex nature of the disease and symptoms^1–3^. According to diagnostic criteria, DLB and PD dementia (PDD) are both defined as Lewy body dementia (LBD) but are distinguished clinically based on the timing of onset of parkinsonism and dementia symptoms^4^. At post-mortem examination these disorders present similar underlying pathological profiles, with, in addition to LBs, mixed pathology of neurofibrillary tangles (NFT) and amyloid beta (Aβ) plaques commonly featuring^5^.

Over the last few years, more studies have been undertaken to investigate the genetic mechanisms underpinning PD^6^ and LBD^7^. These studies have highlighted numerous risk loci associated with these diseases, including 78 loci in PD^6^ and five genome-wide significant loci in LBD, three being known PD risk genes (*SNCA*, *GBA* and *TMEM175*) and two being AD risk genes (*APOE* and *BIN1*)^7^. However, whilst these studies have provided novel insights into disease pathways, genetic variation only explains a small amount of disease susceptibility, with environmental factors also being robustly associated with disease development and progression^8–10^. In recent years, there has been a growing appreciation that epigenetic mechanisms may mediate the interaction between genetic and environmental risk factors in the etiology of sporadic neurodegenerative diseases. Indeed, several epigenome-wide association studies (EWAS) have now been performed in AD, which have culminated in several large-scale meta-analyses, nominating numerous robust and reproducible alterations in DNA methylation in the cortex during disease^11–14^. However, there has been considerably less research exploring DNA methylomic signatures in relation to LB disease, with in fact only one EWAS performed to date with a substantial sample size (*n* > 100) and utilizing multiple independent cohorts^15^. That study utilized the Illumina Infinium MethylationEPIC array to quantify DNA methylation in the prefrontal cortex (PFC) in a primary discovery analysis from 73 control and 249 LB disease spectrum patients from the Netherlands Brain Bank (NBB). Their analysis focused on changes associated with LB pathology development, irrespective of the primary diagnosis and reported 24 loci significantly associated with Braak LB stage, with a strong concordance of the direction of effect with findings from the independent Brains for Dementia Research (BDR) cohort. Among the reported loci, four CpG sites were significant in both cohorts, annotated to *TMCC2*, *SFMBT2*, *AKAP6*, and *PHYHIP*.

To investigate the epigenetic underpinnings of these diseases in relation to neuropathology, we have designed the largest EWAS of LB disease to date, which includes multiple cortical brain regions and a meta-analysis with three independent cohorts. Our analyses utilized 1,205 samples from 832 unique donors and identified 24 false discovery rate (FDR) significant loci associated with LB pathology in the cortex. Leveraging prior studies of DNA methylation undertaken in AD and Huntington’s Disease (HD), we found shared epigenomic profiles between neurodegenerative diseases. Finally, we identified sites with evidence of genetic influence on DNA methylation levels and demonstrated an enrichment of methylation in the *SNCA* gene in LB disease. We observed a strong colocalization of genetic risk loci in the 5’ region of the gene, captured in both LBD and PD GWAS, to cortical methylation quantitative trait loci (mQTLs) for multiple methylation loci.

## Results

### Meta analysis of differential DNA methylation associated with cortical Lewy bodies

**Fig. 1:**
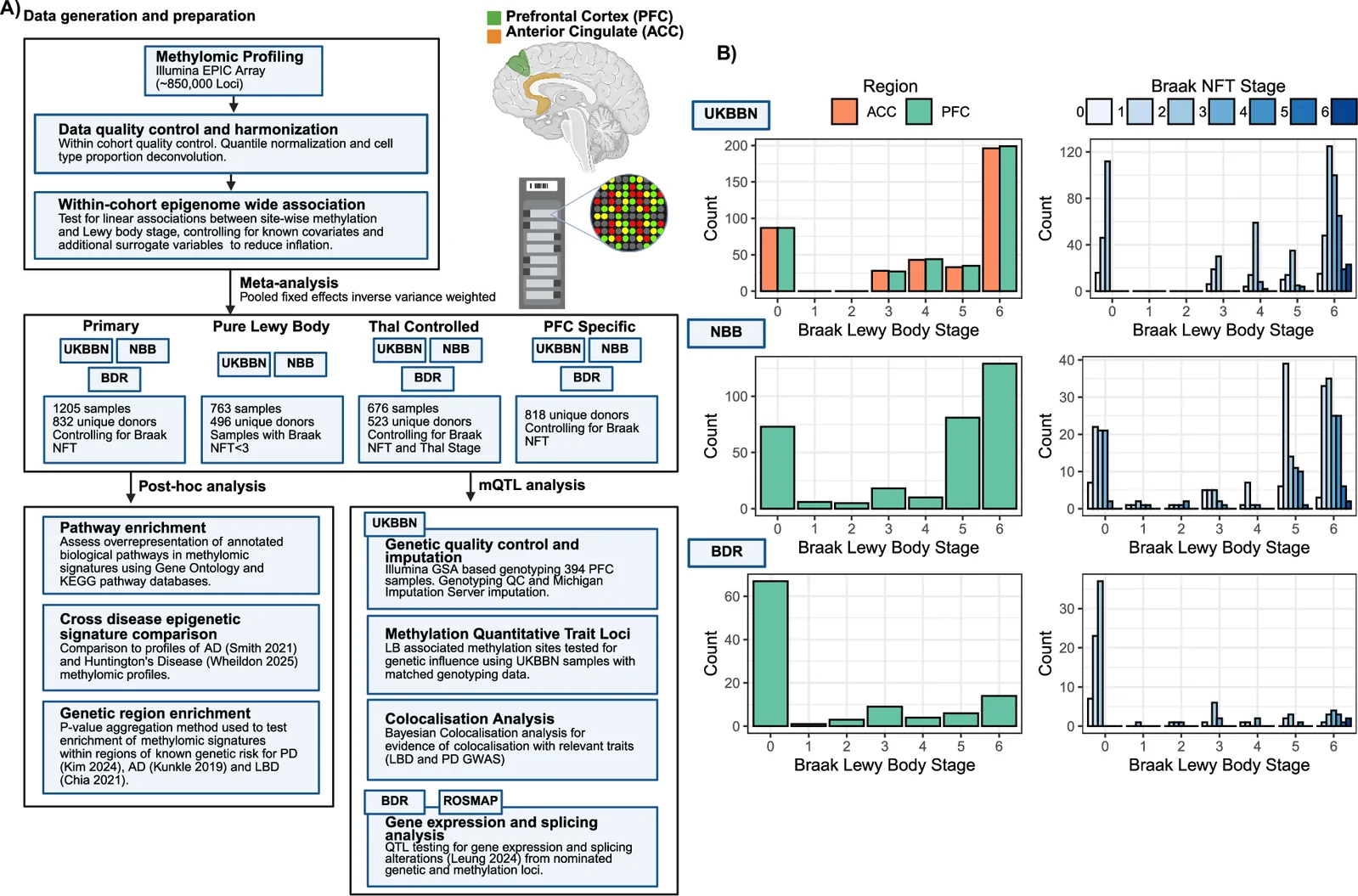
Summary of cohorts and samples used in DNA methylation association meta-analysis of Lewy body pathology. **A** Brain regions and analysis pipeline for meta-analyses. The brain regions include two cortical regions, the prefrontal cortex (PFC, BA9) and the anterior cingulate cortex (ACC, BA24). The analysis plan outlining steps in data generation, harmonization, within cohort linear association analyses and fixed effects meta-analysis. **B** Summary of sample numbers in the UK Brain Bank Network (UKBBN), Netherlands Brain Bank (NBB) and Brains for Dementia Research (BDR) cohorts. Left hand bar plots show number of samples per Braak LB stage, right hand plots show number of samples with coinciding AD pathology as measured by the Braak NFT stage. Components of figure created in Biorender. Harvey, J. (2026) https://BioRender.com/tv5k14q.

**Fig. 2:**
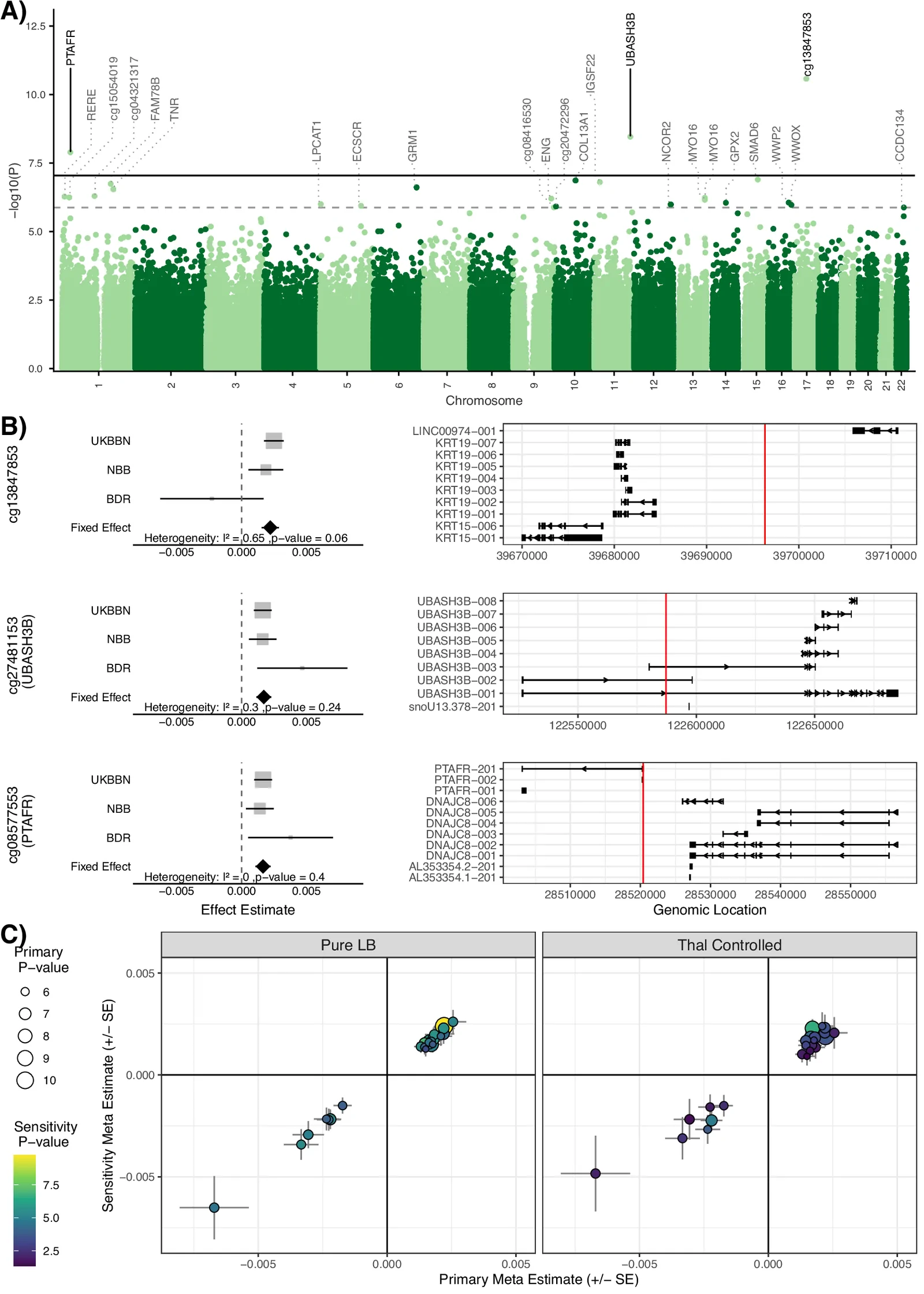
DNA methylation associated with Lewy body Braak stage in a cross cortical meta-analysis. **A** Manhattan plot showing differentially methylated sites associated with Braak LB stage in a fixed effects inverse variance weighted meta-analysis of 1205 total samples (primary full cohort LB pathology meta-analysis). Y-axis shows −log10 transformed *p*-value of association, x-axis shows chromosomal positions (chromosomes 1–22). The black solid horizontal line shows the genome-wide significance threshold (*p* < 9 × 10^−8^), whilst the gray dashed horizontal line denotes the FDR-corrected significance threshold (*p*_adj_ < 0.05). Points are colored to show chromosome number and for those that passed the significance thresholds these are annotated by UCSC gene symbols in the Illumina manifest file or, where unavailable, Illumina CpG ID is displayed. **B** Forest plots of the effect sizes for the three sites passing the genome-wide significance threshold in the primary full cohort LB pathology meta-analysis (total sample size = 1205, unique donors = 832). Gray points show cohort specific estimates (UKBBN: *n* = 779 samples, 406 unique donors; NBB: *n* = 322 donors; BDR: *n* = 104 donors) sized by weight in the inverse variance weighted meta-analysis. Whiskers show 95% confidence interval (CI) of estimate. Diamonds show effect estimates and 95% CI for fixed effect estimates from the primary meta-analyses. The I^2^ heterogeneity statistic and heterogeneity *p*-value are displayed for each site. Genome tracks for each methylation locus (Genome build GRCh37-hg19) are shown to the right, with the location of the associated methylation site marked with a red line. **C** Correlation plot of effect sizes between the primary full cohort LB pathology meta-analysis (y-axis) versus a subset analysis of samples without substantial coincident AD NFT pathology (“Pure LB”, Braak NFT stage ≤II, left plot) or samples where Thal-staging was available to control for concomitant Aβ pathology (Thal-controlled, right plot) (x-axis) for the 24 FDR-significant DMPs identified in the primary full cohort meta-analysis. Points are sized by -log10 transformed *p*-value significance in the primary full meta-analysis and colored by −log10 transformed *p*-value significance in the subset analysis. Effect sizes are displayed as change in methylation (beta value) per increasing LB Braak stage. Error bars show plus and minus the standard error (SE) of each association statistic calculated in each meta-analysis.

Utilizing a dataset comprising three independent cohorts with DNA methylation profiling conducted on the Illumina Infinium MethylationEPIC v1.0 array, we sought to test for associations with the distribution of LB pathology, as measured by the Braak LB staging criteria. The cohort included data generated from the UK Brain Bank Network specifically for this study (UKBBN, *n* = 779 samples, 406 unique donors, *n* = 387 anterior cingulate cortex (ACC), *n* = 392 PFC) and data from two existing datasets: the Netherlands Brain Bank (NBB, *n* = 322 PFC donors)^15^ and Brains for Dementia Research (BDR, *n* = 104 PFC donors)^16^ (Fig. 1). Separately for each cohort, we used linear regression to identify differentially methylated positions (DMPs) associated with LB pathology, including confounding variables (age, sex, cell type proportions, technical batch variables, post-mortem interval and Braak NFT stage) and surrogate variables as required to ensure that the genomic inflation lambda value < 1.1 (Methods, Supplementary Fig. 1). All donors in this dataset were either controls with a pathological confirmation of lacking neurodegenerative disease or required a primary neuropathologically confirmed diagnosis of incidental LB disease, PD, PDD or DLB (Methods). Given that 50-80% of LB disease have concomitant AD pathology^17^ where possible we minimized the number of individuals with advanced Braak NFT stage (>III) in our cohorts based on inclusion criteria for a neuropathological primary diagnosis of LB disease (Fig. 1, Supplementary Data 1, “Methods”). We also included Braak NFT as a covariate in our primary discovery models (Methods). We conducted a fixed effect, inverse variance weighted meta-analysis of the three cohorts (total sample size = 1,205, unique donors = 832) for 774,310 methylation sites passing data quality control (QC). We identified three DMPs associated with LB pathology stage at a genome-wide significance association threshold of *p* < 9 × 10^−^^8^ (Fig. 2A, C), whilst a further 21 DMPs were identified at a more lenient FDR corrected *p*-value threshold (*p*_adj_ < 0.05) (Fig. 2A, Supplementary Data 2). The most significant locus was annotated to probe cg13847853, which is a DMP with previous significant association to AD NFT pathology^14,15^ and has not been previously observed in the context of LB disease. It represents an intergenic locus located between the protein coding genes *KRT19* (−11,777 base-pairs) and *KRT9* (+31,973 base-pairs). It is also proximal to the long non-coding RNA gene *LINC00974* (+9522 base-pairs).

Although we had controlled for Braak NFT stage in our primary meta-analysis, we wanted to further test if coinciding AD related co-pathology could be contributing to our findings. Two sensitivity analyses were performed, the first removing samples with a Braak NFT stage >II (“Pure-LB”, *n* = 763 samples, 496 unique donors) and the second sub-setting the analysis to samples with Thal-staging present and including this as an additional covariate to control for Aβ pathology (“Thal-controlled”, *n* = 676 samples, 523 unique donors). In both instances, this reduced our total sample size and as a likely result, we observed a reduced number of DMPs passing either threshold for multiple testing correction. We observe one site in the Pure-LB analysis (cg13847853 (intergenic): *p* = 1.87 × 10^−10^, Effect = 0.0024, Supplementary Data 3) and two sites in the Thal-controlled analysis (cg14888636 (*MAN1C1*): *p* = 2.78 × 10^−8^, Effect = 0.00303; cg08305436 (intergenic): *p* = 5.44 × 10^−8^, Effect = −0.00305, Supplementary Data 4) associated at the genome-wide significance threshold level. However, despite this, we found that the effect size of the 24 FDR significant DMPs we had identified in the primary full cohort analysis were highly correlated with the effect size from these secondary subset cohort analyses (Pure-LB: Pearson’s correlation (*r*) = 0.998, *p* = 3.8 × 10^−29^, Thal-controlled: Pearson’s correlation (*r*) = 0.983, *p* = 9.35 × 10^−18^), with a 100% concordance in the direction of effect in both cases (Fig. 2C). Standard error estimates were also highly comparable between the primary and subset sensitivity analyses (Pure-LB: Pearson’s *r* = 0.998, *p* = 7.97 × 10^−29^; Thal-controlled: Pearson’s *r* = 0.99, *p* = 1.4 × 10^−20^). All sites prioritized in the primary meta-analysis also retained a nominal association (*p* < 0.05) in the secondary sensitivity analyses (Supplementary Data 5). Together this indicates that the associations identified in the primary full cohort analysis are not dependent on the presence of concomitant NFT or Aβ pathology, and that the loss of significance in this secondary subset analysis is principally a result of reduced power.

To test brain region specific effects, we next conducted individual EWAS analyses in the matched ACC and PFC regions present within the UKBBN dataset. With the reduced sample size, a single site passed the genome-wide significance threshold in the PFC, specifically cg13847853 (*p* = 6.13 × 10^−9^, Effect = 0.0034), which had been the most significant CpG in our primary meta-analysis (Supplementary Data 6b). No significant sites were identified in the ACC specific analysis (Supplementary Data 6c). We also conducted a PFC specific meta-analysis across all three cohorts (*n* = 818 unique donors), identifying four sites passing the genome-wide significance threshold (Supplementary Data 6a). These sites were annotated to the aforementioned intergenic locus (cg13847853, *p* = 2.53 × 10^−9^, Effect = 0.0025), *UBASH3B* (cg27481153, *p* = 5.96 × 10^−9^, Effect = 0.0021), *SMAD6* (cg22230400, *p* = 1.59 × 10^−8^, Effect = 0.0023), and *GRM1* (cg05523624, *p* = 2.14 × 10^−8^, Effect = 0.003). All of these sites had passed FDR significance in our primary cross-cortical meta-analysis, and all 24 FDR significant sites from the primary cross-cortical meta-analysis showed a strong correlation of effects size in the PFC specific meta-analysis (Supplementary Fig. 2).

### Post-hoc analyses highlights enrichment in neuronal processes and identify differentially methylated regions

**Fig. 3:**
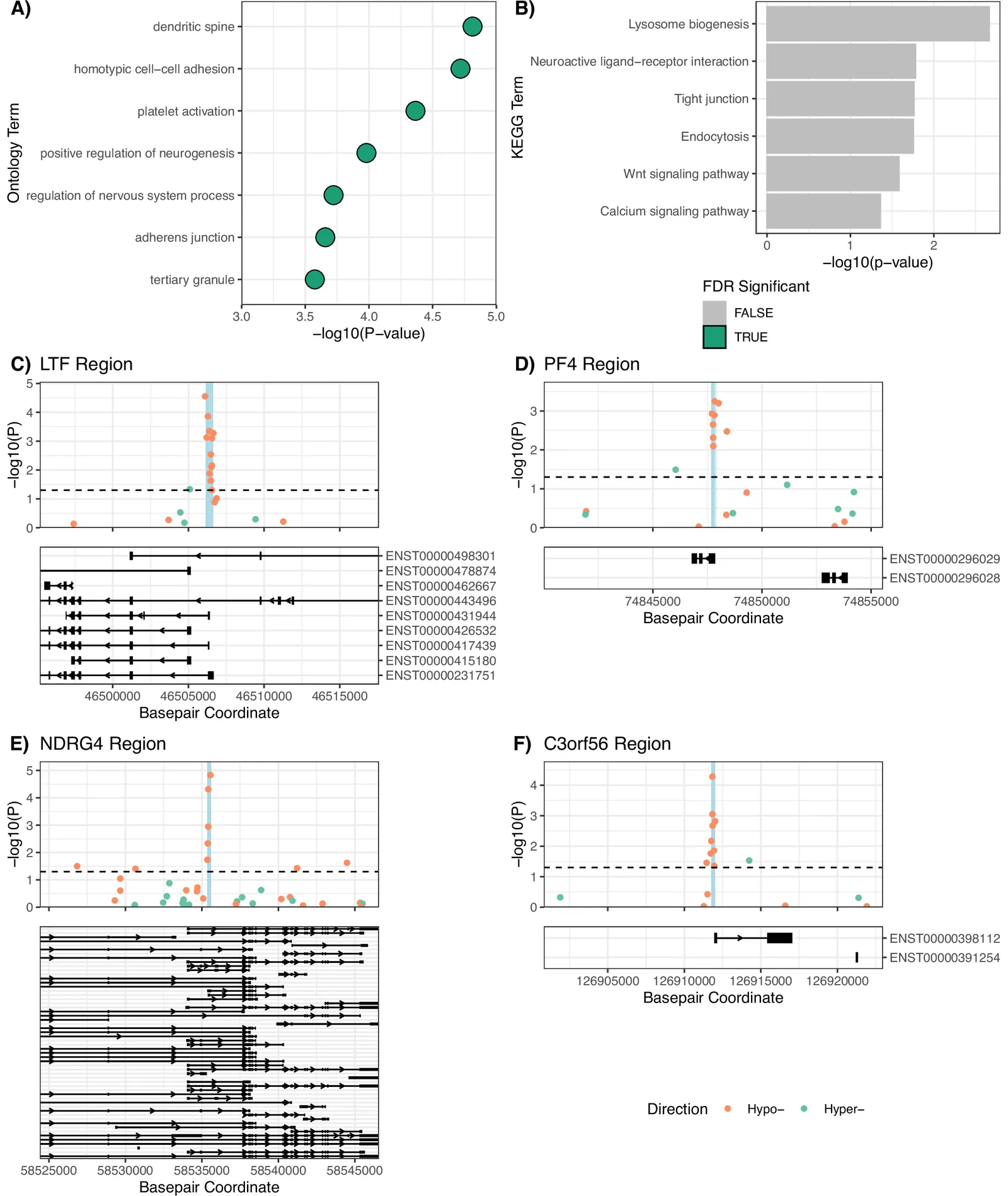
Pathway and regional analysis for the primary full cohort Lewy body pathology meta-analysis. **A** Gene ontology (GO) analysis revealed seven FDR-significant terms, which are shown, with -log10 transformed raw *p*-value shown on the x-axis. REVIGO prioritized gene parent terms are shown on the y-axis. **B** KEGG pathway analysis revealed no FDR significant term. Shown are all nominally significant enrichments with -log10 transformed raw *p*-values shown on the x-axis. **C–F** Mini-Manhattan plots showing the four significant formal DMRs. Shown are **c** the *LTF* gene region, **D** the *PF4* gene region, **E** the *NDRG4* gene region and **F** the *C3orf56* gene region. The y-axis shows -log10 transformed *p*-value for the primary full cohort LB pathology meta-analysis and the x-axis shows the base-pair position. Points show probe coverage for the EPIC array and are colored by their direction of effect in association with LB Braak stage (hypomethylation = orange, hypermethylation = green). The highlighted blue region shows the differentially methylated region (DMR) identified from comb-p analysis. Below track shows the annotated transcripts for the region. The black dashed horizontal line represents nominal significance (*p* < 0.05).

Next to determine potential biological function underpinning the methylation signature identified in our primary full cohort LB pathology meta-analysis, we employed pathway enrichment analysis, using the methylRRA function within the methylGSA package^18^, which aggregates methylation around gene annotations to provide a set of prioritized genes to be tested for over representation analysis using robust rank aggregation (RRA) (Supplementary Data 7). This identified a set of seven FDR-significant enriched terms (Fig. 3A, Supplementary Data 8a), including a number of terms relevant to neuronal and synaptic function (dendritic spine, positive regulation of neurogenesis and regulation of nervous system process), inflammation (tertiary granule) and broader cellular processes (e.g., platelet activation, homotypic cell-cell adhesion and adherens junction). The KEGG pathway analysis did not show any FDR-significant enriched pathways, although we note the topmost nominal pathway was annotated to lysosomal function (*p*-value = 0.0022, *p*_adj_ = 0.095, Fig. 3B, Supplementary Data 8b).

To test for evidence of enrichment of prioritized DNA methylation loci from the primary full cohort meta-analysis in known genomic regions, we used the *knowYourCG* package^19^. We did not find any evidence of specific enrichment in any of the annotated structures within the genome when we tested either the 24 FDR significant loci, or the 67 loci that passed the suggestive significance threshold (*p* < 1 × 10^−5^).

Next, we explored if any genes contained statistically formally defined differentially methylated regions (DMRs), consisting of multiple adjacent DMPs by performing comb-p analysis^20^ on the summary statistics from our primary full cohort LB pathology meta-analysis. We identified four significant DMRs (Supplementary Data 9) overlapping the genes *LTF* (Fig. 3C, Chr3:46506206-46506519, Šidák corrected *p*-value = 6.71 × 10^−9^), PF4 (Fig. 3D Chr4:74847710-74847829, Šidák corrected *p*-value = 5.10 × 10^−6^), *NDRG4* (Fig. 3E, Chr16:58535416-58535556, Šidák corrected *p*-value = 4.73 × 10^−6^), and *C3orf56* (Fig. 3F, Chr3:126911830-126911953, Šidák corrected *p*-value = 1.58 × 10^−4^).

### Exploring cortical LB associated methylation changes in the context of other neurodegenerative diseases

Next, we sought to investigate the disease specificity of the LB associated DMPs we had identified in our primary full cohort LB pathology meta-analysis by comparing these results to summary statistics in previously reported EWASs in AD and HD brain samples. For AD associated effects, we utilized the summary statistics from a prior meta-analysis of NFT pathology in the cortex^14^, performed on the Illumina Infinium Methylation 450 K array and encompassing data from 1408 individuals. For HD associated effects, we used a recently generated case-control cohort of 41 individuals and encompassing matched EPIC array data from the entorhinal cortex (*n* = 38) and striatal (*n* = 37) brain regions^21^.

**Fig. 4:**
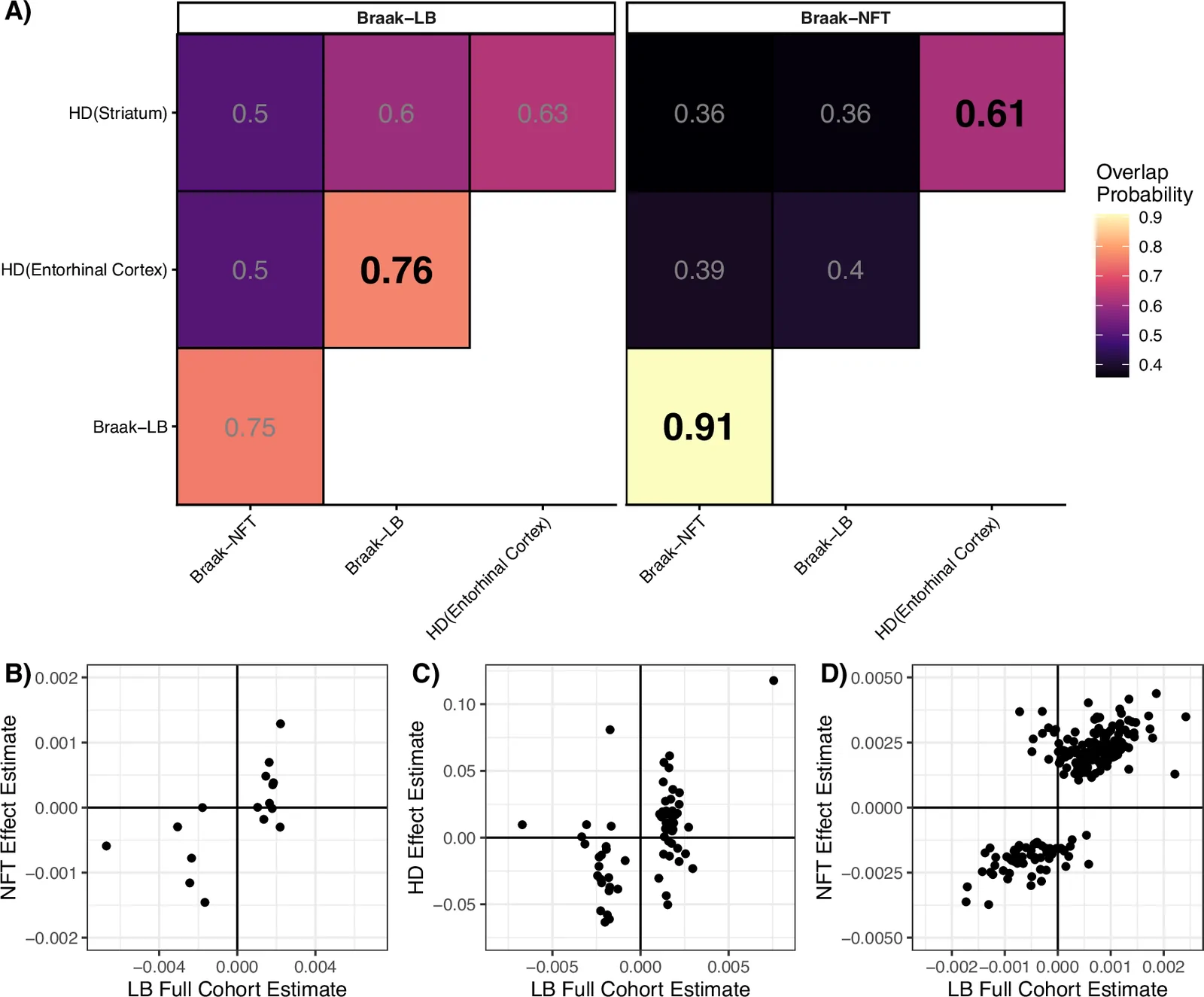
Summary of comparisons of epigenomic signatures between different pathologies. **A** Heatmap of overlap in direction of effect for the most significant LB-Braak associated sites (*p* < 1 × 10^−5^, left panel) and NFT-Braak associated sites (*p* < 1.24 × 10^−7^, right panel) with the effect sizes for different neurodegenerative disease outcomes. The number displayed and fill color are the overlap probability for the same direction of association between two overlapping summary statistics as calculated by the binomial sign test (one-sided). Bolded tiles indicate an FDR significant test result for significant overlap in directionality of associations. Braak NFT effects are taken from Smith et al.^14^ and HD effects from Wheildon et al.^21^. **B**–**D** Scatterplots of DNA methylation associated with different pathologies. **B** Shown for the top LB-associated loci is the comparison of effect size in cortex for LB and NFT (Smith et al.^14^) for the 16 overlapping sites across summary statistics, and **C** for LB and HD (Wheildon et al.^21^) for the 67 overlapping sites across summary statistics. **D** shows the comparison of effect size in cortex for previously reported NFT associated loci (Smith et al.^14^) in our LB samples for the 208 sites overlapping across summary statistics.

Taking the effect sizes for the Braak-LB associated DMPs from our primary full cohort LB pathology meta-analysis at a suggestive significance threshold (*p* < 1 × 10^−5^, *n* = 67 loci), we observed a significant overlap in the direction of effect with HD-associated changes in the entorhinal cortex (Fig. 4A, C, Supplementary Data 10a, binomial test probability = 0.76, *p*_adj_ = 6.51 × 10^−5^), with 51 of the 67 CpGs assessed across both studies showing the same direction of effect. We found a nominally significant overlap with the direction of effect to Braak NFT associated changes for the 16 CpGs that overlapped between the studies (i.e., present across both array platforms) (Fig. 4A, B, Supplementary Data 10a, binomial test probability = 0.75, *p* = 0.038, *p*_adj_ = 0.092), with 12 of the common 16 CpGs showing the same direction of effect. The lack of FDR significant overlap in this binomial test may in part be due to a lower number of overlapping CpGs between the EPIC and 450 K array for testing. However, when we assessed the Pearson’s Correlation measure for direction of effect comparisons between the Braak-LB and Braak-NFT meta-analyses results, as prioritized by the nominally significant Braak-LB CpGs, we did observe a significant positive correlation (Correlation Coefficient = 0.63, *p*_adj_ = 0.02, Fig. 4B).

When we took the Bonferroni significant Braak NFT associated DMPs from the Smith et al. study (*p* < 1.24 × 10^−7^, *n* = 208 loci present on the EPIC array), we found a significant overlap in the direction of effects to Braak-LB associated changes at these CpG sites (Fig. 4A, D, Supplementary Data 10b, binomial test probability = 0.91, *p*_adj_ = 1.26 × 10^−35^), with 189 of these 208 sites showing the same direction of effect. However, we did not observe a significant overlap in the direction of effect for the 207 NFT-associated sites present in the HD study with HD-associated changes in the entorhinal cortex (Supplementary Data 10b). We did however observe a significant overlap in the direction of effect for the 207 CpGs in the HD entorhinal cortex and HD striatum samples (Supplementary Data 10b, binomial test probability = 0.61, *p*_adj_ = 0.00266), with 127 sites showing the same direction of effect. Notably, neither the Braak-LB DMPs, nor the Braak NFT DMPs showed any significant overlap in the direction of effect with the midbrain HD-associated effects in the striatum (Supplementary Data 10a, b), indicating effects are cortex specific. Together, this suggests a shared neurodegenerative methylation signal in the cortex across these three primary pathologies.

However, although a shared cortical methylation signature is apparent across neurodegenerative diseases from our comparison of direction of effects, it is worth noting that the top-ranked loci are distinct across the different diseases. Indeed, for the 24 FDR significant LB-associated DMPs in our primary full cohort meta-analysis, only cg13847853 was Bonferroni-significant in the Braak NFT meta-analysis performed by Smith et al.^21^. Outside of this overlapping locus, no other sites from the primary full cohort LB meta-analysis even showed suggestive significance (*p* < 1 × 10^−5^) in either the Braak NFT or HD EWASs (Supplementary Data 11) and similarly, aside from cg13847853, none of the Bonferroni DMPs from the Braak-NFT meta-analysis showed suggestive significance (*p* < 1 × 10^−5^) in the primary full cohort LB pathology meta-analysis, nor in the HD EWASs (Supplementary Data 12). This indicates that the most robust cortical DMPs in each neurodegenerative disease are specific to that disease.

### Methylation quantitative trait loci analysis prioritizes sites under genetic influence but does not support overlap with known genetic risk loci

A number of studies have shown that DNA methylation can be influenced by genetic variation, commonly termed mQTLs. Given the size of our cohort, we utilized matched genotyping array data for 386 PFC samples from the UKBBN cohort to test for mQTLs at the 24 FDR significant methylation sites nominated in our primary full cohort LB pathology meta-analysis. We identified 530 FDR significant cis-mQTLs annotated to eight methylation loci: cg09966204 (*MYO16*), cg15054019, cg07837822 (*MYO16*), cg27625119 (*LPCAT1*), cg22516725 (*WWOX*), cg21912448 (*RERE)*, cg05523624 (*GRM1*) and cg25194322 (*FAM78B*) (Supplementary Data 13, Supplementary Fig. 3). We further identify 68 significant trans-mQTLs annotated to three methylation loci: cg06441533 (*GPX2*; *CHURCH1-FNTB*), cg13650687 (*COL13A1*) and cg20472296 (Supplementary Data 13). However, none of the single nucleotide polymorphisms (SNPs) corresponding to the identified cis-mQTLs overlapped with GWAS hits reported in summary statistics from GWAS of AD^22,23^, PD^6^ or LBD^7^.

### The *SNCA* region shows enrichment of differentially methylated loci and evidence of colocalization with genetic risk signatures

To test if genomic regions prioritized by GWAS of AD^23^, PD^6^ and LBD^7^ show enrichment for LB pathology associated methylation, we employed Brown’s method to combine p-values (from the primary full cohort LB pathology meta-analysis) across the disease-associated linkage disequilibrium (LD) regions (Supplementary Data 14). Across the combined 99 regions from the three GWASs tested, only the *SNCA* region, as annotated in the recent LBD (Chr4: 90743331 – 91005096, *p*_adj_ = 0.037) and PD (Chr4: 89835093–91453046, *p*_adj_ = 0.037) GWASs showed significance after multiple testing correction. Exploring this region in more detail, we saw that six loci in the *SNCA* gene showed evidence of a nominal association (uncorrected *p* < 0.05), all residing in the 5’ region of the gene and showing hypomethylation with increasing LB Braak stage (Supplementary Fig. 4).

**Fig. 5:**
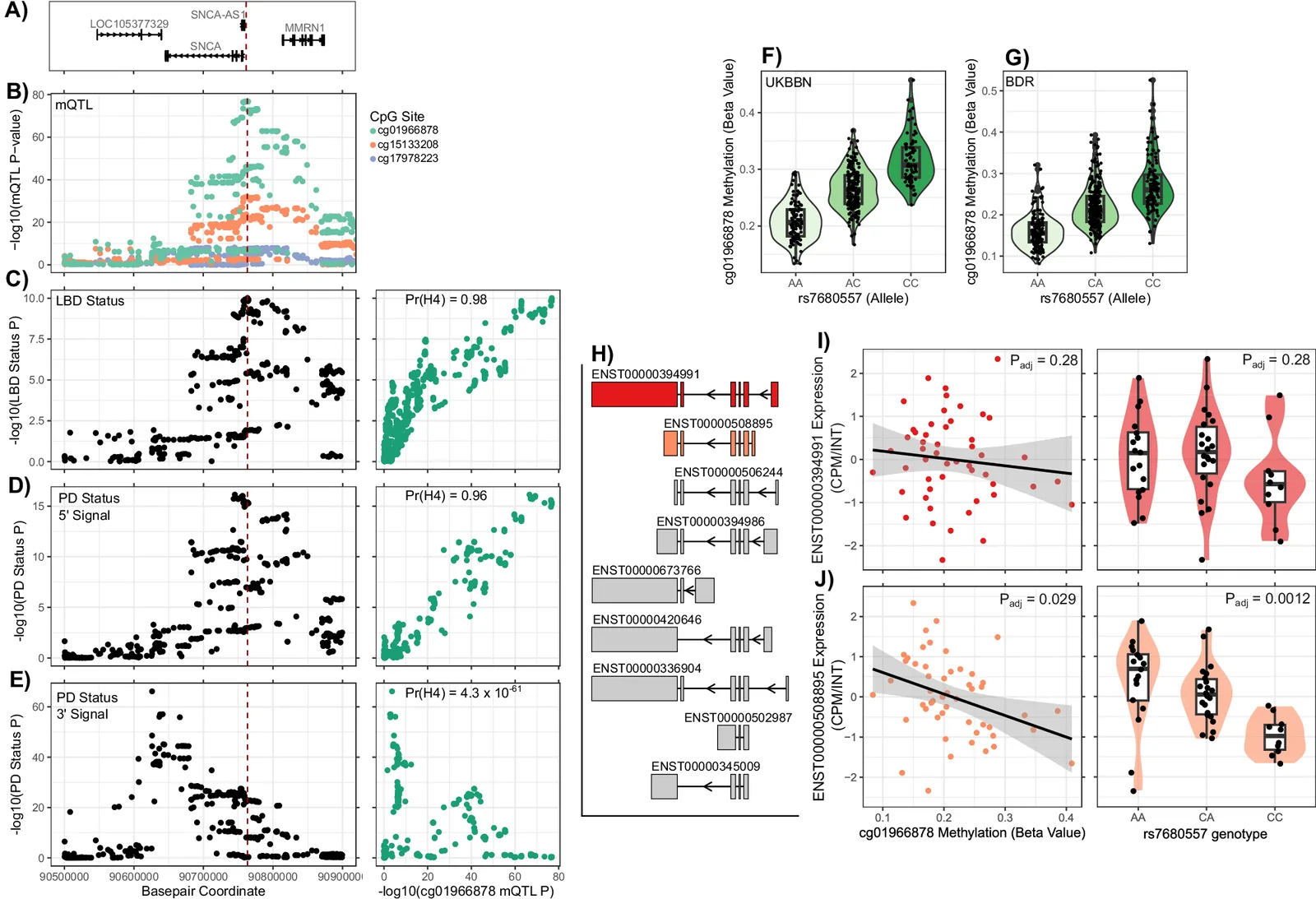
Characterization of *SNCA* methylation quantitative trait loci. **A** Genomic tracks showing the *SNCA* coding region and surrounding genes (Build 37, hg19). Shown below the track are the same genomic regions with mini-manhattan plots showing the associations of the following traits: **B** mQTL associations for the three methylation sites with evidence of colocalization with LB-relevant GWAS. Points are colored by cpg annotation for mQTL association, **C** GWAS for Lewy Body Dementia (Chia et al.^7^), **D** Conditioned GWAS for Parkinson’s Disease (PD) (Pihlstrom^72^), results represent associations controlling for the lead SNP in the 3’ *SNCA* region (rs356182). **E** Conditioned GWAS for PD (Pihlstrom et al.^72^), results represent association controlling for the secondary associated signal in 5’ *SNCA* region (rs763443). On the right of each GWAS plot is a correlation plot displaying the -log10 transformed *p*-values for that GWAS on the Y-axis and the -log10 transformed *p*-values for the strongest mQTL signature (annotated to cg01966878) on the X-axis. The colocalization prior probability (Pr(H4)) is shown within each plot, with a value closer to 1 indicative of high probability of colocalization under a shared causal variant. Within each mini-manhattan plot is a red-dashed vertical line annotating the lead-SNP location for LBD (rs7680557). **F**–**G** Box and violin plots showing lead-LBD SNP rs7680557 genotype and DNA methylation at cg01966878 in the UKBBN (*n* = 386) and BDR (*n* = 523) datasets. **H** Tracks representing all called transcript isoforms of *SNCA* identified in the previously generated dataset in BDR (*n* = 49) (Leung et al.^25^). Highlighted in red is the highest expressed canonical transcript of *SNCA* (ENST00000394991) and highlighted in orange is the genotype and methylation associated alternative isoform (ENST00000508895). Shown to the right is the association with lead-LBD SNP rs7680557 and methylation at cg01966878 for **I** the canonical and **J** alternative transcripts. *P*-values represent linear association model results (detailed in Methods). All boxplots displays the median as a central line, the first and third quartiles as hinges and the whiskers display the minimum and maximum range (excluding outlying points greater that 1.5 times the interquartile range). Line on the scatterplots displays a linear model based line with 95% confidence interval shown as a shade.

Given our findings of enriched epigenetic alteration in the *SNCA* region, we next tested if the methylation sites in the region show evidence of QTL relationships with genetic variation in this region using the UKBBN cohort. We identified 16 methylation sites in this region that showed significant evidence of a cis-mQTL relationship including five of the six CpG sites nominally associated with LB pathology in the primary methylation meta-analysis (Supplementary Data 15). Notably, the strongest mQTL relationship observed was to cg01966878 (Fig. 5A, B), which was the most strongly associated site overlapping *SNCA* in the primary full cohort LB pathology meta-analysis (*p* = 2.06 × 10^−4^, Effect Estimate = −0.0034).

We next tested if the mQTL profiles in the *SNCA* region and the genetic association signatures of GWAS for LBD and PD show evidence of colocalization under shared causal variants. The mQTL signal for cg01966878 showed strong evidence of colocalization to both LBD genetic risk (Fig. 5C, Colocalization Pr(H4) = 0.98) and the 5’ secondary conditional PD genetic signature (Fig. 5D, Colocalization Pr(H4) = 0.96), but not to the strongest PD genetic risk signature located 3’ of the gene (Fig. 5E, Colocalization Pr(H4) = 4.3 × 10^−61^). Evidence of colocalization was also observed at two additional CpG sites (cg15133208 and cg17978223, Supplementary Data 16). We observed that the most significant mQTL relationship of cg01966878 to the LBD risk locus rs7680557 identified in UKBBN (Fig. 5F, Effect Estimate = 0.053, *p* = 1.63 × 10^−77^) is also reproduced in the BDR cohort (Fig. 5G, Effect Estimate = 0.054, *p* = 7.39 × 10^−69^). We highlight that the direction of effect between the SNP and DNA methylation at cg01966878 is concordant with the expected direction from the original GWAS and methylation meta-analysis findings. That is, the A-allele, which is associated with increased risk of LBD, is strongly associated with hypomethylation, which in turn is the direction we observed in association with increased Braak LB-stage (Supplementary Fig. 4).

Using available reference data from the ROSMAP cohort^24^, we tested the association of both the genetic locus (*n* = 504) and the top prioritized methylation locus on *SNCA* gene expression (*n* = 526). When assessing short-read RNA sequencing data, we find no evidence of an effect of either rs7680557 or cg01966878 on *SNCA* transcript expression (Supplementary Fig. 5). In line with previously reported QTL relationship with the *SNCA*-antisense (*SNCA-AS*1) transcript, we observe evidence of association of rs7680557 to *SNCA-AS1* expression (Effect estimate with C-allele = 0.15, *p*_adj_ = 0.04), (Supplementary Fig. 5), with consistent direction of effect as prior reports^7^. No significant association was observed between DNA methylation at cg01966878 and *SNCA-AS1* expression after accounting for multiple comparisons (*p*_adj_ = 0.16, Supplementary Fig. 5d).

Although, we had not observed an association at the overall transcript level, we next tested if splicing isoform associations could be detected with rs7680557 or cg01966878 by leveraging a long-read RNA sequencing dataset of 49 samples that had been previously generated in the BDR cohort^25^ (Fig. 5H). A *SNCA* transcript resulting in differing 5’ and 3’ untranslated region (UTR) structure (ENST00000508895) was significantly associated with both the LBD risk variant rs7680557 (Fig. 5J, *p*_adj_ = 0.0012) and the mQTL prioritized cg01966878 methylation site (Fig. 5J, *p* = 0.029). Consistent with the short-read RNA sequencing data, we did not observe any evidence of association with the canonical primary transcript isoform for *SNCA* (ENST00000394991) and either rs7680557 (Fig. 5I, *p*_adj_ = 0.28) or cg01966878 (Fig. 5I, *p*_adj_ = 0.28).

## Discussion

In this study, we present a meta-analysis of cortical DNA methylation associated with LB pathology. We find evidence of significant differential methylation at 24 loci across the genome, the top three of which reached the most stringent cutoff for genome-wide interpretation (i.e., cg27481153 (*UBASH3B*), cg08577553 (*PTAFR*) and cg13847853). We find evidence that the functional annotations enriched in prioritized loci are related to a number of brain relevant traits including neuronal and synaptic function. Comparison of our results to previous EWASs of AD-related neuropathology (Braak NFT) and HD status indicates a shared epigenomic profile across some, but not all, neurodegenerative diseases. Specifically, when taking top prioritized sites from the primary full cohort LB meta-analysis, we find evidence of significant shared directional effect with changes reported in HD cortex as well as nominally significant shared directionality with cortical AD profiles. Alternatively, when taking sites nominated from a prior Braak NFT meta-analysis, we find evidence of shared directionality specifically with this meta-analysis, but with no evidence of concordance with HD profiles. When assessing known GWAS regions associated with LB-diseases, we find evidence of hypomethylation in the *SNCA* region and when looking at mQTL relationships, find evidence of strong colocalization with disease associated genetic variation located at the 5’ region of the gene. We further provide evidence that these changes associate with the expression of an alternative *SNCA* transcript isoform, but not the primary canonical transcript.

A number of the genes prioritized from the primary full cohort LB meta-analysis have been previously related to LB-related traits. *UBASH3B* encodes Ubiquitin Associated and SH3 Domain Containing B, which is highly expressed in brain tissue and has a variety of related functions in cell signaling^26^. It has been previously associated in PD and DLB contexts, reported as differentially expressed in single cell analyses of prefrontal cortex astrocytes^27^ and inhibitory neurons of the anterior cingulate cortex^28^. Rare variants in the gene have also been associated with serum total tau levels in an exome sequencing study^29^. *PTAFR* encodes Platelet Activating Factor Receptor, highly expressed in brain tissue and implicated in cell signaling via the NF-κB pathway. It has been reported to be differentially expressed in AD hippocampal tissue^30^ and is implicated in mediating astrocytic inflammatory responses^31^. cg13847853 is a more perplexing site in terms of functional annotation but has been previously associated with AD NFT related pathology^14^. It resides between a long non-coding RNA (*LINC00974*) and the gene *KRT19*. *KRT19* is minimally expressed in brain, although it has been associated on a proteomic level in the periphery to Aβ pathology^32^. Further work is required to elucidate the downstream regulatory impact of this methylation site.

Of the FDR significant differentially methylated loci, a number have been previously associated with parkinsonian and LB related traits. *WWOX*, encoding WW Domain Containing Oxidoreductase, is a gene which is linked to autosomal recessive neurodevelopmental disorders^33^, but has also been shown in large GWAS to be associated with both AD status^23^ and the development of cognitive decline in PD^34^. We note that an FDR-significant site annotated to the gene *GRM1*, encoding glutamate metabotropic receptor 1 also lies downstream of the gene *RAB32* which has been recently identified as a causal gene in autosomal dominant PD^35^.

To address the potential for confounding AD-related NFT pathology, we utilized a secondary meta-analysis restricted solely to samples with minimal co-pathology as defined by Braak NFT staging. This reduced the total viable sample size and, perhaps unsurprisingly, resulted in far fewer sites passing multiple testing correction. Solely cg13847853, the topmost associated loci in our primary full cohort LB pathology meta-analysis, retained genome-wide significance. Similarly, when we restricted the analysis to samples with Thal staging information available and controlled for this as a covariate in meta-analysis as a method of addressing Aβ related pathology, loci at *MAN1C1* and cg08305436, both not detected in our primary full cohort LB pathology meta-analysis, reached genome-wide significance. Notably however, there was a strong concordance in estimated effects between the primary and sensitivity meta-analyses, which we interpret as evidence that the LB-stage associated effects from our primary analysis are not contingent on the presence of coincidental NFT and Aβ pathology.

Our post-hoc testing implicated epigenetic changes in the *SNCA* gene, despite this region not passing the multiple testing threshold used in our primary or secondary meta-analyses. In a targeted assessment of the gene, we found evidence of hypomethylation in a number of sites in the 5’-region of the gene, which we believe warrants highlighting given that multiple other studies have reported the same phenomena in relation to LB disease^36–40^. Furthermore, when employing a method to aggregate *p*-values from our meta-analysis results based on genetic regions reported from related GWAS, we found that the only significantly enriched region corresponded to the *SNCA* region reported in the most recent PD^6^ and LBD^7^ GWASs. We find evidence that a number of methylation loci at the 5’ region of the gene shows strong evidence of mQTL relationships. The most significant mQTL relationship observed in this region showed evidence of colocalization and shared causal variants to the genetic risk profile of LBD and the secondary, conditioned genetic risk signature of PD. This observation is in line with previous work^41^ and provides evidence that genetic variation in the 5’ region of *SNCA* is relevant to functional alterations in the cortex, in-keeping with findings in relation to both LBD and REM sleep behavior disorder^7,42^. Although we do not find evidence of expression alterations in total *SNCA* expression resulting from implicated genetic or epigenetic loci, we do report evidence of isoform specific associations in *SNCA*. Specifically, ENST00000508895 was associated with both the lead genetic risk locus for LBD and our identified mQTL. This transcript does not result in alterations in the peptide sequence of *SNCA* but is altered in its 5’ and 3’ UTR compared to the canonical *SNCA* transcript isoform. ENST00000508895 has also been previously reported as an LBD-risk specific QTL in an independent dataset^43^. More work is warranted to determine the functional implications of this transcript. Given that *SNCA* differential methylation has a reported cell-type specific effect^44^, future studies looking at cell type specificity of DNA methylation in *SNCA* may yield sites in the gene at genome-wide significance.

We further find evidence of a shared epigenomic signature across multiple neurodegenerative diseases and pathologies. There was a clear comparability between epigenetic effects for LB and NFT pathologies in the cortex, indicating a level of shared molecular processes related to both pathologies. However, notably only cg13847853 was associated at genome-wide significance in both analyses, indicating that despite a shared profile between the two, the prioritization of individual processes may differentiate the different pathologies. This shared effect is consistent with other modalities of genetic and biological data, with genetic risk in genes such as *CLU*, *WWOX* and *GRN*^45^, as well as the *HLA* region^46^ having been shown to have associated overlap between PD and AD and evidence of a level of shared transcriptomic alteration in DLB and AD brains^47^. Perhaps more surprisingly was evidence found here between LB and HD associated epigenomic profiles, specific to the cortex. This shared effect did not appear to be indicative of a broad neurodegenerative signal in the cortex, as no evidence of comparability was seen between HD and NFT pathological profiles. Similarly, we did not find evidence of shared profiles with HD associated changes in the striatum. Given that HD is caused by a highly penetrant autosomal dominant genetic defect and shares minimal pathology with LB disease, this finding is perplexing. One potential explanation is that both diseases are marked by a shared degeneration of the striatal regions and the similar cortical epigenomic profiles may represent a secondary signature of midbrain neurodegeneration. Another explanation may be that as the disease progresses, HD cortical profiles show similar underlying epigenetic profiles to those observed in the vulnerable striatal regions. Further work is needed to determine if this shared epigenomic profile we observed between these differing movement disorders represents a potential shared biological etiology or secondary effects to other common features of the two conditions. We note that the comparability of effects sizes between studies may be influenced by power of the initial EWAS used to prioritize associated sites. In the case of the HD study, which had a notably smaller sample size than either of the meta-analyses of NFT or LB related pathology, this may limit the ability to detect shared methylomic signatures. Further work, in singular study designs incorporating multiple neurodegenerative diseases at a consistent power, is needed to fully elucidate the contribution of shared epigenomic signatures to multiple neurodegenerative diseases.

We found evidence that some of the FDR significant methylation sites identified in our primary meta-analysis have evidence of genetic influence from our mQTL calling. However, we found no evidence that the genetic variants prioritized from this analysis are associated with neurodegeneration related phenotypes from previous GWAS. This is in-keeping with similar findings from previous meta-analyses of neurodegenerative pathologies which have failed to find evidence of pleiotropy between AD genetic risk variants and mQTLs associated with primary EWAS prioritized methylation sites^14^.

One limitation of our study is the quantified neuropathological outcomes used to determine association and control for confounding covariance. In this analysis we have used Braak LB stage, due to its use in previous studies of this type both for LB disease and AD^15^. Although Braak LB stage is a useful instrument in assessing PD-relevant pathology progression, its utility in the broad context of LB diseases, in particular DLB, is highly questioned. Indeed, alternate staging schemes focus more on categorically subtyping pathological LB by its distribution in different brain regions^48^ as opposed to an ordinal semi-quantitative scheme that assumes pathology initiates in the brainstem. Thus, in our analysis, samples which are grouped into specific Braak LB stages may present highly variable LB pathology burden in the cortical regions we are assessing. This decision was chosen for practicality to allow for a harmonized measure between this study and previous EWASs. Braak LB staging was also the most represented pathological staging scheme in the brain bank records that we had access to. Similarly, although we made efforts to stratify and control for AD co-pathology as measured by Braak NFT stage as this was available for all samples, we could not control for Aβ pathology for all samples used. This is because measurements of Aβ deposition, such as the Thal stage, were absent for a large proportion of available cases. We did seek to address this potential confounder when we performed the lower powered sensitivity EWAS that included Thal stage as a covariate however, further work is needed to fully determine the effect of Aβ on LB-associated epigenomic profiles. One challenge when studying post-mortem brain tissue in neurodegenerative diseases is the impact of neuronal cell loss, and gliosis on cell populations, which may impact the DNA methylation signatures observed in disease. Although we have attempted to mitigate this by controlling for the proportions of different neuronal and glial cell types, future analyses should be undertaken on sorted cell populations in order to identify cell-type specific signatures. Differences in the cell type deconvolution model used in this study, which resolves five cellular proportions, compared to less-granular models which resolved fewer cell types employed in previous studies, may also account for differences in results with previous EWASs in LBDs^15^. Notably, a recent study from our group testing our LB meta-analysis results with a tool for measuring enrichment of cell specific epigenetic signatures reported enrichment of our LB DNA methylation signature in microglial and oligodendrocytic cells^49^.

In summary, we have undertaken the largest meta-analysis of DNA methylation in LB diseases to date, highlighting robust methylation alterations at 24 loci, and highlighting epigenetic alterations in *SNCA* in disease. Future studies warrant DNA methylomic analyses of well-characterized cohorts with detailed ante-mortem and post-mortem clinical and pathological assessments, in order to identify signatures that distinguish between different LBDs that show similar pathological profiles with more distinct clinical symptoms. The integration of DNA methylation data with other epigenetic marks and transcriptional profiling will allow the identification of disease signatures that impact at multiple layers of genomic regulation and may represent unexplored candidates when developing pharmacological intervention strategies.

## Methods

### Cohort descriptions

Samples were sourced from the UK Brain Bank Network (UKBBN), an online database covering brain bank centers across the UK and annotated with demographics, neuropathological and clinical assessments. All available records on the UKBBN were downloaded (June 2019) for manual selection. First, to determine LB positive case availability, records were filtered on the basis of neuropathological diagnostic criteria. Only cases with an annotation of Braak LB stage of 3–6, or annotation of neocortical or limbic LB pathology were selected as LB disease cases. The majority of samples matching our search criteria were found in brain banks at Oxford, Newcastle, Imperial College London and University College London. Samples were sourced on the basis of a primary diagnosis of either PD or DLB, either in available clinical records or at post-mortem examination. Samples were included if they had a primary neuropathologically confirmed diagnosis of PD or DLB and, where primary diagnosis was absent, had a “High” assessment of the likelihood that the pathological profiles were associated with typical DLB clinical syndrome from the 2017 Lancet criteria^4^. Exclusions were made for familial synucleinopathies or misdiagnosed primary symptoms (e.g., multiple system atrophy (MSA), Progressive Supranuclear Palsy (PSP), etc.), severe cerebrovascular disease or TDP-43 pathology, as well as removing cases annotated with amygdala predominant LB pathology. Seventy-one cases had annotation for LB disease relevant pathology following the 2009 Unified staging scheme but lacked Braak LB staging annotation^50^. To harmonize these annotations for analysis alongside the Braak scheme, records were converted in accordance with the BrainNet Europe Consortium^51^. Sixty-one cases with “Neocortical Lewy Body Disease” were annotated as Braak LB stage 6. The remaining ten cases showed evidence from the available additional neuropathology notes of LB pathology in the temporal cortex or cingulate gyrus and were annotated as Braak LB stage 5. From this criterion, we selected 28 cases with Braak LB stage 3, 44 cases with Braak LB stage 4, 36 cases with Braak LB stage 5 and 208 cases with Braak LB stage 6. An additional 90 control individuals without neurological disease were sourced, which were chosen to exclude significant cerebrovascular pathology or substantial AD-related pathology (Braak NFT stage >II). A set of 20 samples lacked explicit Braak NFT stage in their record but had sufficient tau staining reported in the neuropathology records for Braak NFT stage to be imputed. In total, a set of 406 total donors from UKBBN were chosen for our study, with two brain regions sourced for the majority of individuals. These brain regions were the ACC (Brodmann Area (BA) 24) and the PFC (BA9). All samples received for our study had informed consent at local brain banks, covered under individual ethical agreements at the center (REC references 24/NE/0012, 15/SC/0639, 18/LO/0721, 23/WA/0273). Ethical approval for our study was granted from the University of Exeter Medical School Research Ethics Committee (13/02/009). This study complied with all relevant regulations regarding the use of human study participants and was conducted in accordance to the criteria set by the Declaration of Helsinki.

Data for the Netherlands Brain Bank (NBB) cohort has been previously detailed in Pihlstrøm et al., where they had analyzed PFC tissue from 73 controls, 29 incidental LB disease, 139 PD (of which 74 were PDD), and 81 DLB patients^15^. Data from the BDR Cohort was previously detailed in Shireby et al.^16^. Neurological controls were selected with absence of LB-pathology and low AD co-pathology (Braak NFT < III), along with the absence of significant neuropathological co-pathology (TDP-43 pathology, gross infarction, severe cerebrovascular disease). 20 samples overlapped between the UKBBN and BDR cohort and were removed from the BDR cohort prior to analysis. Case samples were selected in keeping with the same criteria as the UKBBN. A final set of 104 samples from the BDR cohort were used for the meta-analysis.

### Sample preparation and methylomic profiling

For the UKBBN cohort, tissue pieces ranging between 25 and 35 mg were cut from frozen PFC or ACC tissue before being milled over liquid nitrogen. Following tissue grinding, DNA was extracted using the Qiagen AllPrep DNA/RNA/miRNA Universal kit according to the manufacturer’s instructions and stored at 4 °C. 500 ng of DNA from each sample was bisulfite treated using the Zymo EZ-96 DNA methylation Gold kit. Methylomic profiling was completed after sample randomization using the Illumina Infinium MethylationEPIC v1.0 BeadChip.

### DNA methylation analysis, quality control and normalization

Data processing was performed using R, and signal intensity files were loaded into R using the minfi package^52^. All data was subjected to a thorough quality control (QC) pipeline using the minfi^52^ and wateRmelon packages^53^. A MethylumiSet object was created from iDATs using the readEPIC function from wateRmelon^53^ and an RGChannelSet was created using the minfi package^52^. Samples were excluded from further analysis for the following reasons: (1) they had a median methylated or unmethylated sample intensity <1500 or <1000, respectively, (2) that the bisulfite conversion efficiency was <80%, (3) there was a mis-match between reported and predicted sex, (4) if the sample was determined to be an outlier using the outlyx function or, (5) if the samples were found to be cryptically related to one another or unrelated to its matched sample from the other brain region. Finally, using the pfilter function in the WateRmelon package^53^ samples with a detection *p*-value >  0.05 in >5% of probes, probes with <three beadcount in 5% of samples and probes having 1% of samples with a detection *p*-value > 0.05 were also removed. Following QC, samples not passing the aforementioned checks were removed and quantile normalization was performed using the dasen function in the wateRmelon package^53^. Beta values were extracted for further analysis. Cell type proportion calculations were implemented using the projectCellTypeWithError() function within the CETYGO package^54^, using the reference panel resolving enriched profiles for GABAergic neuron, glutamatergic neuron, oligodendrocyte, microglia and astrocyte reference data^55^. To explore whether there may be any potential cell type confounding, cell proportion was tested against LB Braak stage in each cohort (Supplementary Data 17, Supplementary Fig. 6). Two cell type proportional changes passed FDR multiple testing correction, both in the ACC region of the UKBBN data, with a significant increase in the microglia enriched proportion (NeuN^-^/Sox10^-^/IRF8^+^, *p*_adj_ = 0.018, Estimate = 0.0035) and a decrease in the oligodendrocyte enriched proportion (NeuN^-^/Sox10^+^, *p*_adj_ = 0.046, Estimate = −0.01). No significant associations, even at nominal threshold, were detectable for the PFC region in any of the cohorts tested.

### Within cohort epigenome wide association assessment

Prior to meta-analysis, the association with LB Braak stage was assessed within each cohort in a harmonized approach. Within each cohort, linear models were fit using the base lm() function in R to test for associations between LB Braak stage and DNA methylation at each site of the genome. This is displayed by the following equation for the brain region specific UKBBN, NBB and BDR analyses:

$${CpG}\,{Site}\, \sim 	 \, {Braak}\,{LB}\,{Stage}+{Age}+{Sex}+{PMI}+{Cell}\,{Proportion} \\ 	+{Batch}+{Braak}\,{NFT}\,{Stage}\ldots$$

For the UKBBN cross cortical analysis, given two brain regions were present per individual, this caused a violation of linear regression by non-independence between samples type. To correct for this, a mixed effect model was employed using the lme() function within the nlme package. Brain region was included as a fixed effect covariate and individual ID was supplied as a random-intercept covariate to account for paired observations. This can be displayed in the following formula:

$${CpG}\,{Site}\, \sim 	 \, {Braak}\,{LB}\,{Stage}+{Age}+{Sex}+{PMI}+{Cell}\,{Proportion} \\ 	+{Batch}+{Braak}\,{NFT}\,{Stage}+(1|{Individual})\ldots$$

Consistent covariates of age, sex, post-mortem interval (PMI), cell proportions and Braak NFT stage were included in all three cohorts. For the UKBBN and BDR cohorts, brain bank source and array plate batch were included as processing batch variables. For a small subset of the UKBBN donors, PMI values were missing (*n* = 12) and in these cases, mean imputation was used to fill missing values. This method was justified based on previous data exploration for best methods to deal with missingness in phenotypic data^56^. For NBB, only processing plate batch was included as an additional covariate. For each cohort, model *p*-value inflation was assessed genome-wide and surrogate variables added to reduce the lambda value to a <1.1 threshold. One surrogate variable was included for BDR and three were included for NBB, with no surrogate variables required to reduce inflation in the UKBBN cross cortex and PFC analyses. For the UKBBN ACC specific analysis, three surrogate variables were included (Supplementary Fig. 1).

### Meta-analysis

Estimated regression coefficients and standard errors were extracted from each model and used for meta-analysis of all probes overlapping each dataset (*n* = 774,310). Harmonized models were meta-analyzed using the metagen function in the meta R package^57^, as had been employed for previous EWAS meta-analyses^14,15,58^, which applies an inverse variance weighting. Both fixed and random effects were assessed, with the fixed effects model being the primary interpreted results, given the number of cohorts available and the observation that the majority of top sites identified did not show evidence of significant heterogeneity (Supplementary Data 2–4). In addition to fixed and random effects modeling statistics (Effect estimates, standard error and *p*-values), the I^2^ heterogeneity measure and corresponding *p*-values were also extracted.

For interpretation of results, a strict genome-wide significance threshold was based on a previous simulation and permutation testing study to define a significance level as determined by Mansell and colleagues^59^. This approach sets a consistent significance threshold of *p*-value < 9 × 10^−8^ for any study utilizing the Illumina EPIC array and was determined based on multiple permutations of null EWAS study scenarios to determine a 5% family wise error rate. A less stringent multiple testing FDR significance threshold (*p*_adj_ < 0.05) was also used in our study. Finally, for more exploratory post-hoc analyses, a more lenient suggestive threshold was applied of uncorrected *p*-value < 1 × 10^−5^.

### Differentially methylated region analysis

To identify differentially methylated regions (DMRs) from summary statistics from our meta-analyses, we employed the combined *p*-values method as implemented in the python package comb-p^20^, which identifies DMRs based on a defined sliding window, correcting for spatial autocorrelation between adjacent methylation sites. We performed this with the default settings for consistency with prior meta-analyses, with a distance of 500 base-pairs (bp) and a seeded *p*-value of 1 × 10^−4^. Significant DMRs were defined as those with a Šidák’s multiple testing correction *p*-value < 0.05 and with ≥two methylation sites residing in the identified DMR. For plotting, EnsDb.Hsapiens.v75 and GenomicRanges were used to extract annotated transcripts for genome build 37 hg19.

### Pathway enrichment and genomic feature analyses

Pathway enrichment analysis querying the overlap of our findings for enrichment in either the Gene ontology (GO) or Kyoto Encyclopedia of Genes and Genotypes (KEGG) databases was performed using the package methylGSA function methylRRA^18^. This method assesses genome-wide summary statistics, represented by *p*-values, and aggregates them around gene annotations, controlling for the number of CpGs annotated to different genes. This method defined a set of 187 genes (Supplementary Data 7), with evidence of significantly aggregated DNA methylation changes within the primary full cohort meta-analysis summary statistics that were taken forward for enrichment analysis. We utilized the overrepresentation analysis approach, which applies a within gene Bonferroni correction and an across gene FDR correction to prioritize genes for ontological enrichment analyses. Given the overlap of similar ontology terms, for interpretation of significant enriched terms in our results, we further employed the REVIGO method, as implemented in the rrvgo R package^60^ using default parameters to identify shared parent terms for closely related GO annotations.

To test for evidence of enrichment of prioritized DNA methylation changes in known genomic regions, the *knowYourCG* package^19^ was implemented using the packages EPIC array datasets relevant for CpG Island context, histone marks and transcription factor binding sites. We tested both the FDR significant sites and sites that passed the suggestive significance threshold (*p* < 1 × 10^−5^).

### Cross disease epigenomic signature comparison

Assessment of overlap between EWASs was conducted by assessing the directionality of estimated effects in available summary statistics. To statistically compare shared direction of effects, a one-sided binomial sign test was performed to test for greater than expected overlap in association directionality. For comparison to AD related Braak NFT associated signatures, summary statistics were sourced from Smith et al.^14^, which had performed a cross-cortical meta-analysis^14^. This had utilized 1,408 unique donors, which had been profiled on the Illumina Infinium Methylation 450 K BeadChip. For assessment of HD-associated signatures, summary statistics were sourced from Wheildon et al.^21^. This included matched data for 38 entorhinal cortex and 37 striatal samples, profiled on the Illumina EPIC array. The Braak NFT EWAS signature comprised the 208 sites on the EPIC array corresponding to the 220 sites passing Bonferroni significance in Smith et al.^14^. For the Braak LB signature, given that more modest *p*-values were identified, we used the 67 sites passing our suggestive *p*-value threshold of 1 × 10^−5^ in the primary full cohort LB pathology meta-analysis. Following all comparisons tested, FDR correction was applied for interpreting the significant overlap in shared direction of association.

### Genotyping

For the UKBBN data, genotyping was conducted using the Illumina Global Screening Array, which assays >500,000 common SNPs. Raw iDAT files were imported into Illumina Genome Studio 2.0 and converted to PLINK binary format. QC was performed using PLINK (v1.9)^61^, excluding SNPs with >5% missingness, samples with >2% missingness, SNPs with a minor allele frequency (MAF) < 0.001, and Hardy-Weinberg Equilibrium (HWE) *p* < 1 × 10⁻⁵. Samples were also checked for concordance between reported and genetic sex, triallelic SNPs were removed, and runs of homozygosity were assessed.

Genotype data were merged with 1000 Genomes Project Phase 3 (GRCh37 build)^62^ and restricted to autosomal variants, with triallelic variants and allele mismatches removed. Following harmonization, variants were filtered (MAF ≥ 0.2; genotype missingness ≤ 1%) and linkage disequilibrium pruning was performed using a sliding window approach (window size = 50 variants, step size = 5 variants, *r*² threshold = 1.5). A genetic relationship matrix was constructed using GCTA (v1.93.2beta)^63^, and principal component analysis was performed on the combined dataset (study samples and 1000 Genomes individuals), with samples of non-European ancestry excluded from downstream analyses.

Genotype data passing QC were prepared for imputation using PLINK 2.0^64^, restricting analyses to autosomal variants (chromosomes 1–22). Allele frequencies were calculated to support downstream harmonization. Variant identifiers, genomic positions, and alleles were aligned to the Haplotype Reference Consortium using the HRC-1000G-check-bim pipeline^65^. Harmonized genotype data were converted into a unified VCF file using BCFtools (v1.9)^66^ and compressed using bgzip prior to imputation via the Michigan Imputation Server^67^. Post-imputation QC excluded variants and samples with high missingness (>20% and >2%, respectively), variants with MAF < 0.01, and variants deviating from HWE (*p* < 1 × 10⁻⁵). Heterozygosity rates were calculated using an LD-based sliding window (50 SNPs, step size = 5 SNPs, *r*² threshold = 0.2), and cryptic relatedness was assessed, removing samples with *π* > 0.2. After post-imputation QC, 401 individuals overlapping the DNA methylation dataset and 1,149,592 variants remained for downstream analyses.

For the BDR cohort, imputed data was sourced, as previously described^68^.

### Methylation quantitative trait loci analysis

Methylation quantitative loci (mQTLs) were identified in the UKBBN data using a set of 386 samples with matched Illumina Global Screening Array (GSA) genotyping data and PFC DNA methylation data. mQTLs were tested for the 24 loci at FDR significance in the primary full cohort LB pathology meta-analysis. The R package matrix eQTL was used to identify methylation sites with significant association to genetic loci^69^. Methylation beta-values were tested against genotype (coded additively) utilizing a linear model for association testing while adjusting for confounders of age, sex, cell-type proportion estimates and the first three genetic principal components. For the *SNCA* regional analysis, 36 methylation sites annotated to *SNCA* were subset and run as a separate analysis using identical methods to those described above for the 24 FDR nominated loci. Matrix eQTL was run in linear model mode with an additive genetic model to assess cis- and trans-mQTLs. Cis-mQTLs were defined as SNPs located within ±1 Mb of the associated CpG site, while trans-mQTLs correspond to SNPs >1 Mb from the CpG. The significance threshold was adjusted for multiple testing using FDR correction, with a significance threshold of *p*_adj_ < 0.05.

### Genomic enrichment analysis

To test for genomic enrichment in LB associated methylation loci in known regions prioritized by prior GWAS, we employed Brown’s method, as implemented in the EmpiricalBrownsMethod R package to aggregate *p*-values across multiple methylation loci^70^. Regions in linkage disequilibrium (LD) with primary loci in GWAS were extracted for AD, PD and LBD. For PD^6^ and AD^23^, reported regions were taken directly from the supplementary material of the respective study. For LBD^7^, as regions were not directly reported, regions were manually annotated based on the minimum and maximum position of variants at genome-wide significance (*p* < 5 × 10^−8^) in the genome-wide summary statistics around the five primary loci reported in the study. *P*-values were FDR corrected to account for the number of total genomic regions tested. Methylation beta matrices were z-score normalized using the scale() function in R within each cohort, to allow for the correlation calculations within the Brown’s methodology accounting for inter-cohort variability whilst retaining the inter-site correlations.

### Colocalization analysis

We utilized the coloc R package for Bayesian colocalization analysis^71^. mQTL summary statistics were tested for each of the CpG sites in the *SNCA* region separately, overlapping to loci present in both mQTL results and GWAS summary statistics tested. The coloc analysis was run using the full effect estimates, variance estimates, minor allele frequency and *N* values for each summary statistic set. An H4 prior probability of >0.9 was interpreted as evidence of colocalization. Given the *SNCA* genetic risk profile from the largest GWAS of PD to date represent multiple nominated variants, we utilized summary statistics of a conditioned analysis of the region to test the independent effects of the primary and secondary genetic association signals in PD^72^. For this analysis, the results conditioned on the strongest GWAS locus rs356182 were labeled the “PD 5’ Signal”, whilst the results conditioned on the secondary locus rs763443 were labeled the “PD 3’ Signal”. The third independent signal at rs280004 and the unconditioned summary statistics were also tested. For LBD, the largest GWAS to date was utilized^7^.

### SNCA gene expression analysis

To analyze the effect of DNA methylation and genotype at the nominated *SNCA* locus with gene expression, short- and long-read RNA sequencing data from previously published PFC samples were sourced. For the short read expression analysis, genotyping and DNA methylation data from the ROSMAP cohort was sourced covering matched Illumina 450 K methylation array data, and imputed genotype data as described previously^24^. Preprocessed Fragments per Kilobase of transcript per Million mapped reads (FPKM) were sourced for expression analysis. CIBERSORT deconvolution was applied to the FPKM normalized reads to derive reference proportions of neurons, microglia, oligodendrocyte precursor cells (OPC), oligodendrocytes, astrocytes, and endothelial cells. For all testing, to account for non-normal distribution of expression values, FPKM normalized reads underwent inverse normal transformation. *SNCA* and *SNCA-AS1* were extracted and tested for association against genotype at rs7680557 (coded as number of C-alleles, *n* = 504 matched samples) and DNA methylation at cg01966878 (*n* = 526 matched samples) using linear regression analysis. For all analyses, linear models included covariates of age, sex, PMI, RNA integrity number (RIN), batch and CIBERSORT derived proportion estimates^73^. For genotype analyses, the first three genetic principal components were also included in models. For DNA methylation analyses, confounding variables of sex, age, PMI, batch and methylation derived cell proportions were corrected for using multiple regression residuals before testing against expression values.

For assessment of *SNCA* transcript isoforms, previously published data from 49 PFC samples in the BDR cohort with targeted Oxford Nanopore long-read RNA sequencing was sourced^25^. For this analysis, only transcripts with prior annotation were included and were required to fulfill a full-splicing match criteria in SQANTI3 for inclusion. Where multiple isoforms for an individual annotated transcript were present, these were concatenated into a single isoform annotation. Transcript isoforms were counts per million normalized (CPM) using the cpm() function in edgeR, followed by inverse normal transformation. Each called transcript isoform of *SNCA* was tested for its association with DNA methylation at cg01966878 and genotype at rs7680557. For DNA methylation, models controlled for age, sex, batch, RIN, methylation derived cell proportions and the first three genetic principal components. For genotype testing, the first three genetic principal components were also included in testing. FDR correction was applied to account for multiple testing across all genotype, DNA methylation and transcript isoform combinations tested.

### Reporting summary

Further information on research design is available in the Nature Portfolio Reporting Summary linked to this article.

## Supplementary information

Supplementary Information

Description of Additional Supplementary Files

Supplementary Data 1-17

Reporting Summary

Transparent Peer Review file

## Source data

Source Data

## Data Availability

Methylation data and available phenotypic data used in primary analyses for the UKBBN cohort is available on the Gene Expression Omnibus (GEO) Platform under accession number GSE341563. Due to the sensitive nature of raw genotyping data this is available to the community under reasonable request and data sharing agreements. Data for the NBB cohort is available via GEO at accession number GSE203332. Data for the BDR cohort is available via GEO at accession number GSE197305 and additional donor data is available via Dementia Platform UK (https://portal.dementiasplatform.uk/). ROSMAP cohort, genotyping, methylation and expression data is available through the synapse platform (https://www.synapse.org/) under accessions syn7357283 [https://www.synapse.org/Synapse:syn7357283], syn3388564 [https://www.synapse.org/Synapse:syn3388564], syn3157325 [https://www.synapse.org/Synapse:syn3157325] and syn25006903 [https://www.synapse.org/Synapse:syn25006903]. Processed data for relevant primary analyses of DNA methylation cohorts is available for download at: https:// https://zenodo.org/records/20581498. Genome-wide summary statistics for meta-analyses performed in this study are available at https://github.com/UoE-Dementia-Genomics/LB-Meta-Analysis. Source data are provided with this paper.

## Code availability

Analysis scripts used in this manuscript are available at https://github.com/UoE-Dementia-Genomics/LB-Meta-Analysis^74^ (https://doi.org/10.5281/zenodo.20581498).
