# Supplementary material for: Interrogating DNA methylation associated with Lewy body pathology in a cross brain-region and multi-cohort study
Source: Nat Commun. 2026 Sep 15;17:10845. doi: 10.1038/s41467-026-77009-x (PMC13672336; doi:10.1038/s41467-026-77009-x)
Supplement: Supplementary file 1 [file 41467_2026_77009_MOESM1_ESM.pdf]

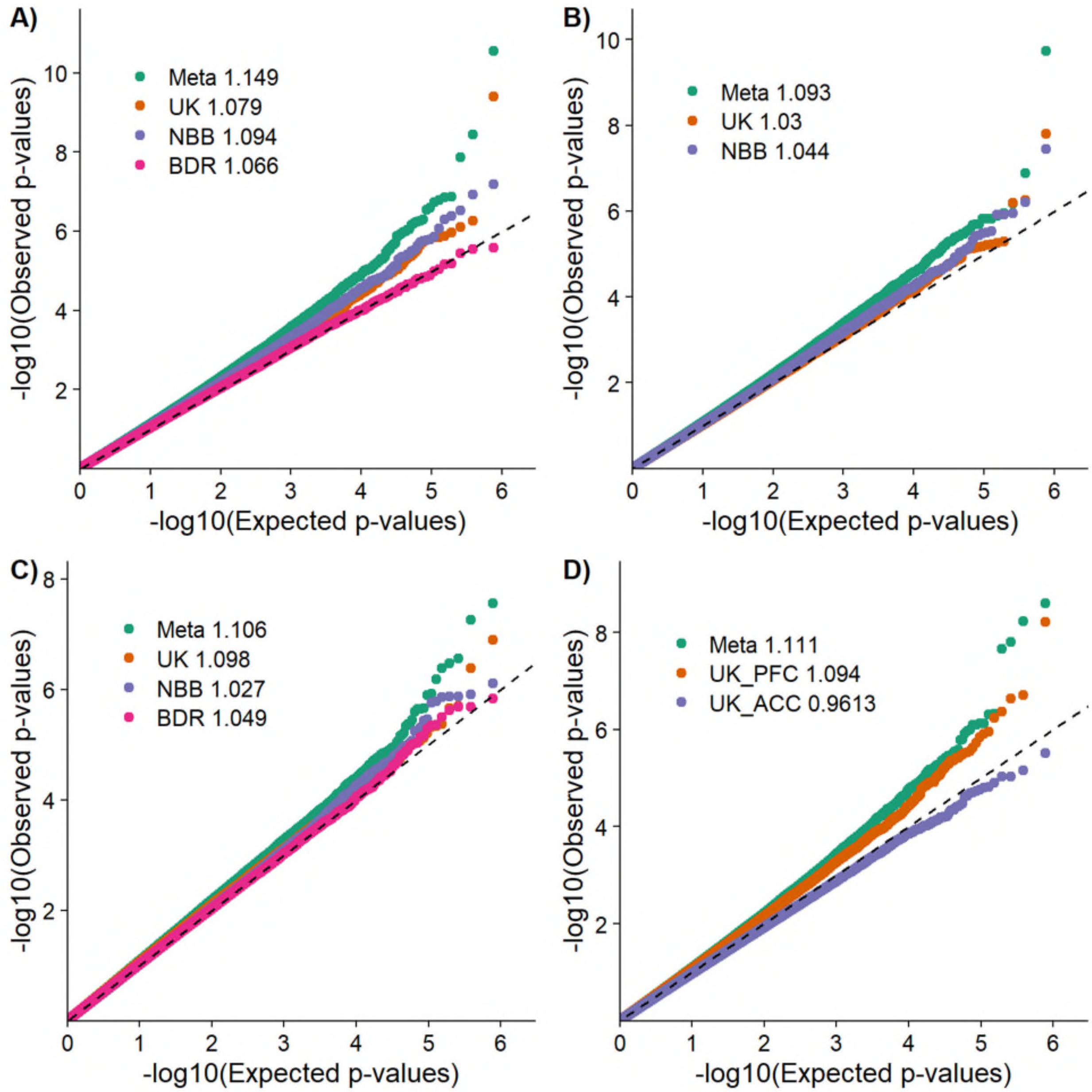


**Supplementary Fig. 1: Quantile-Quantile (QQ) plots showing genomic inflation for each cohort and final meta-analysis fixed effect p-values.** Shown are **a** primary full cohort LB pathology meta-analysis, **b** secondary pure LB pathology subset cohort meta-analysis, **c** Thal-controlled LB pathology meta-analysis and **d** brain region specific EWAS and PFC meta-analysis. Abbreviations: Meta = Meta analysis fixed effect p-values, UKBBN = UK Brain Bank Network mixed effect model p-values, NBB = Netherlands Brain Bank linear model p-values, BDR = Brains for Dementia Research linear model p-values, PFC = Prefrontal Cortex, ACC = Anterior Cingulate Cortex. Shown next to each cohort and test is the Lambda genomic inflation value for each EWAS p-value set tested.

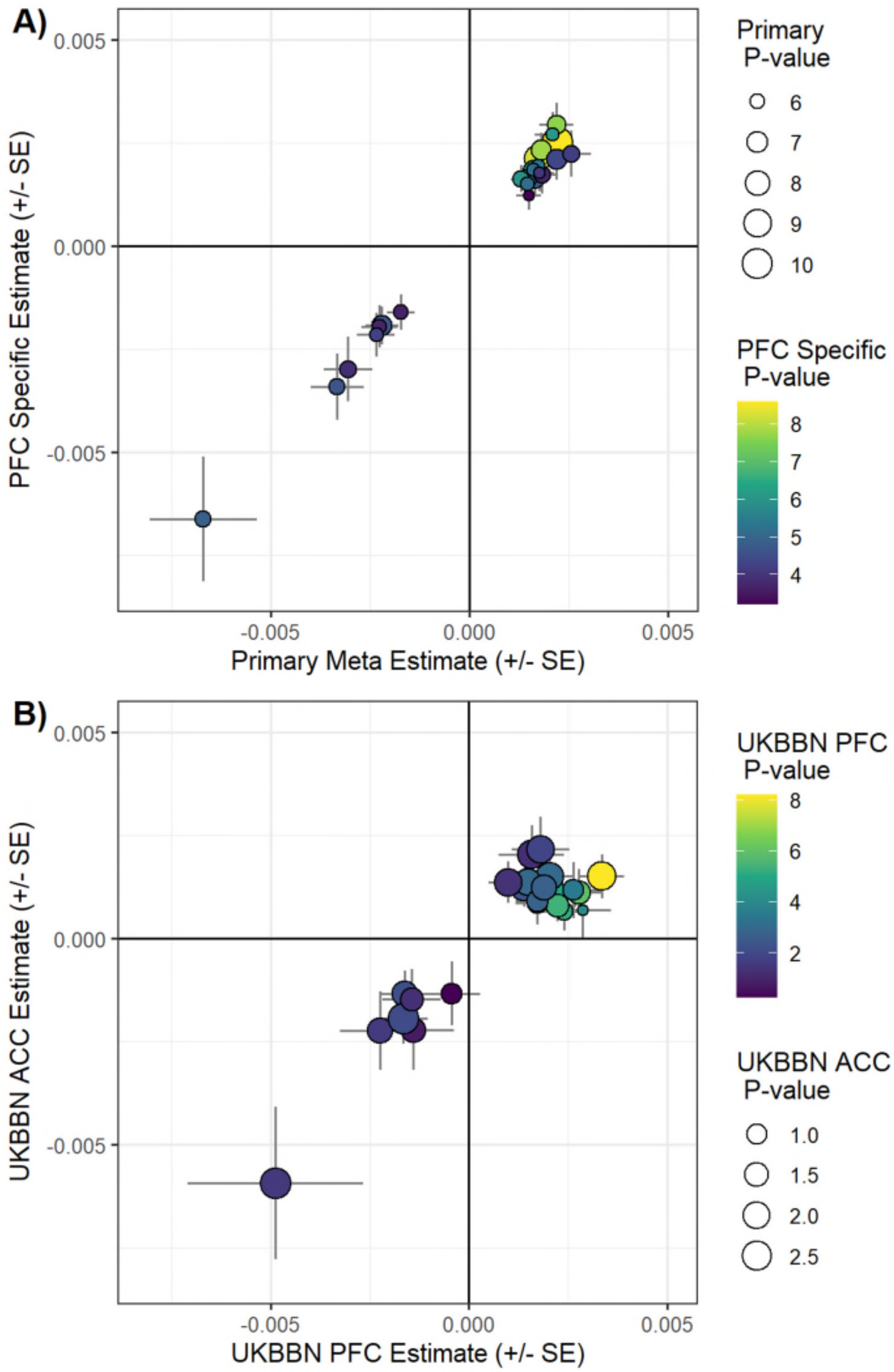


**Supplementary Fig. 2: Effect size correlation plots comparing region specific analyses. a** Comparison between the primary full cohort LB pathology meta-analysis (x-axis) versus the prefrontal cortex (PFC) specific meta-analysis (y-axis) for the 24 FDR-significant DMPs identified in the primary full cohort LB pathology meta-analysis. **b** Comparison between the PFC specific EWAS (x-axis) and the anterior cingulate cortex (ACC) specific EWAS (y-axis) performed in the UKBBN cohort for the 24 FDR-significant DMPs identified in the primary full cohort meta-analysis. For both plots, points are sized by -log10 transformed p-value significance in the primary full meta-analysis and colored by -log10 transformed p-value significance in the subset analysis. Effect sizes are displayed as change in methylation (beta value) per increasing LB Braak stage. Error bars around points show +/- the standard error of the effect estimate for the respective EWAS.

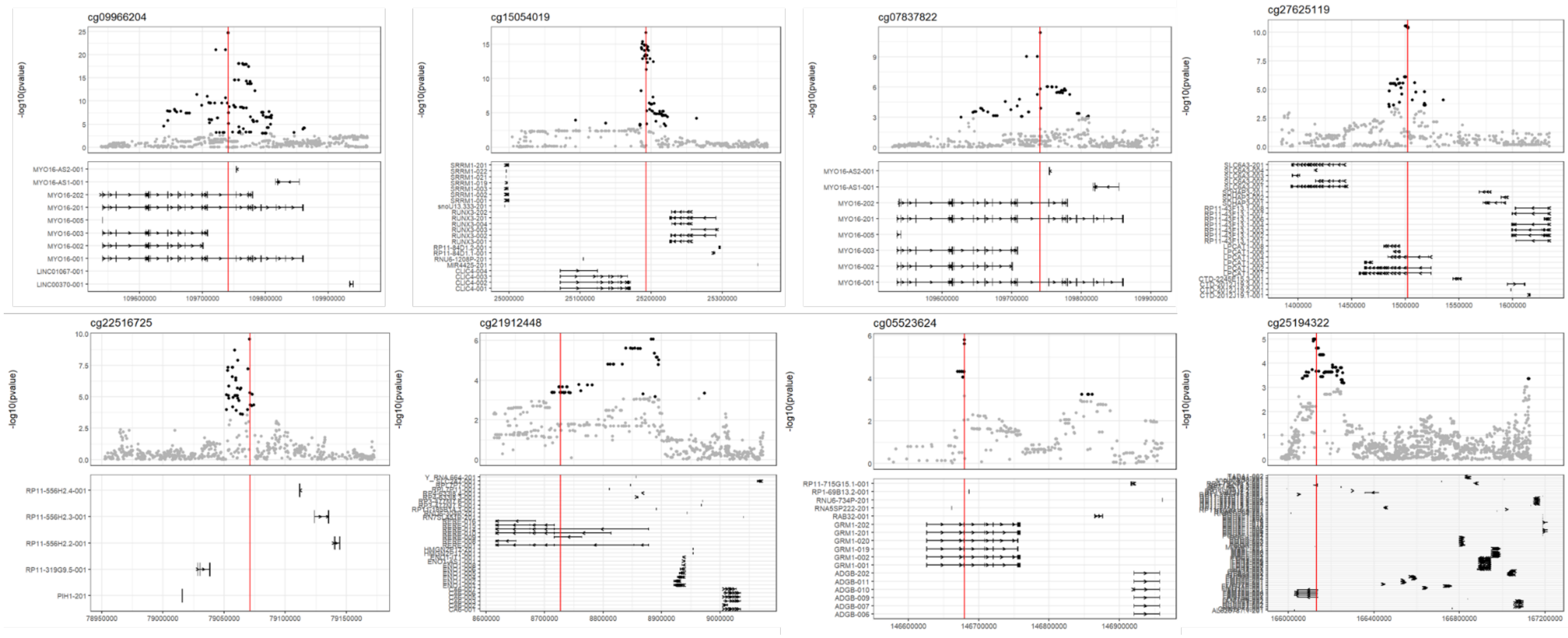


**Supplementary Fig. 3: Significant *cis*-methylation quantitative trait loci (mQTL) relationships in the UKBBN cohort for FDR significant loci identified in the primary full cohort LB pathology meta-analysis.** X-axis shows base-pair coordinate, with y-axis showing unadjusted -log10(p-value) for genotype-methylation associations. Red vertical bar shows location of the methylation site. For each mini-manhattan plot, tracks showing genomic context is shown. Dark points represent FDR-significant mQTL associations with grey points showing non-significant associations. Red vertical line shows the location of the methylation loci in the mQTL relationship.

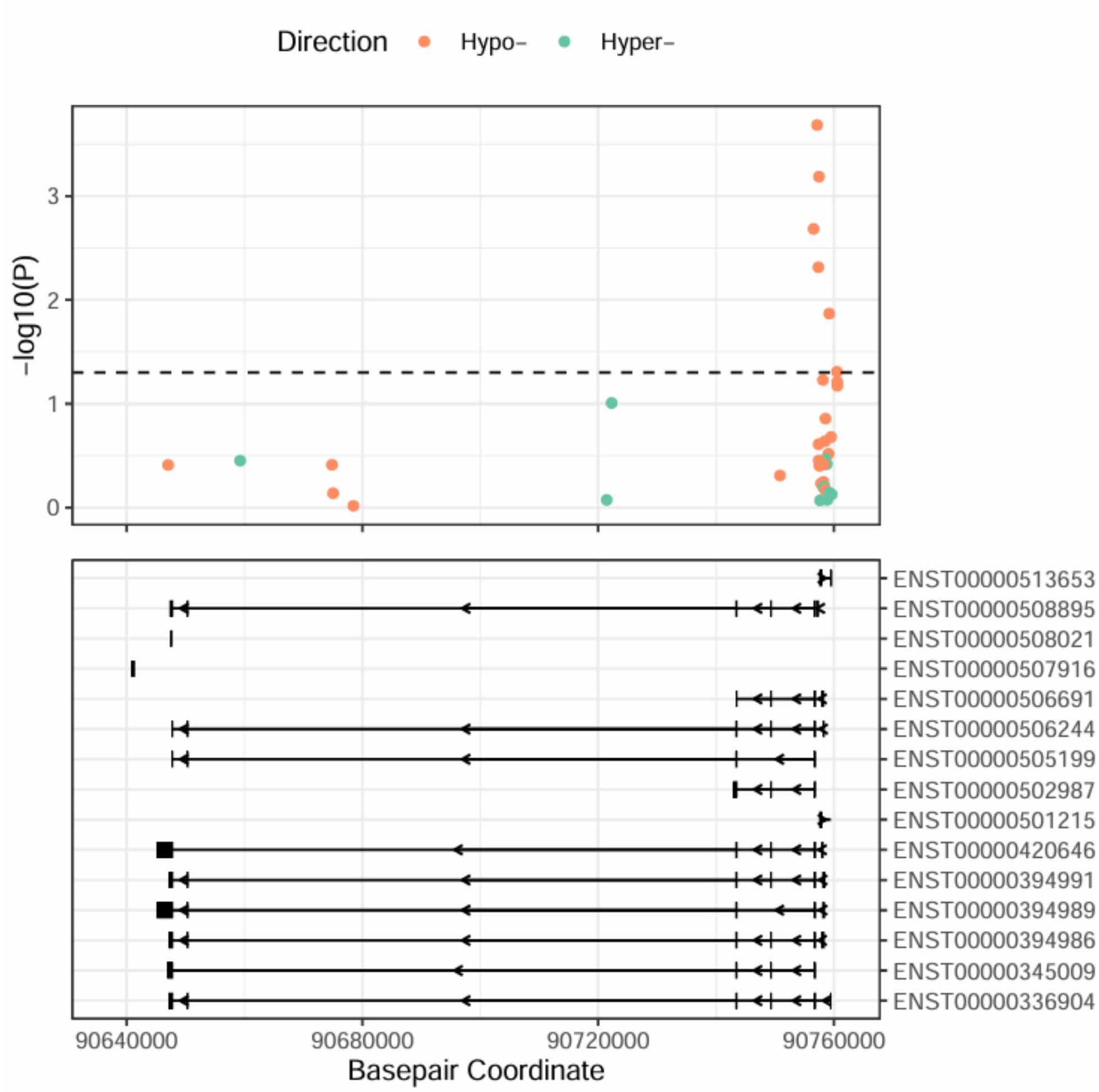


**Supplementary Fig. 4: Six probes in the *SNCA* gene showed nominally significant hypomethylation in the primary full cohort LB pathology meta-analysis.**. Shown are all probes annotated to the *SNCA* gene region (Chr4:90637041-90770556), with those showing hypomethylation in association with LB Braak stage colored in orange, and those showing hypermethylation colored in green. The black dashed horizontal line denotes nominal significance ($p < 0.05$). Underneath the *SNCA* and *SNCA-AS1* gene transcripts are annotated.

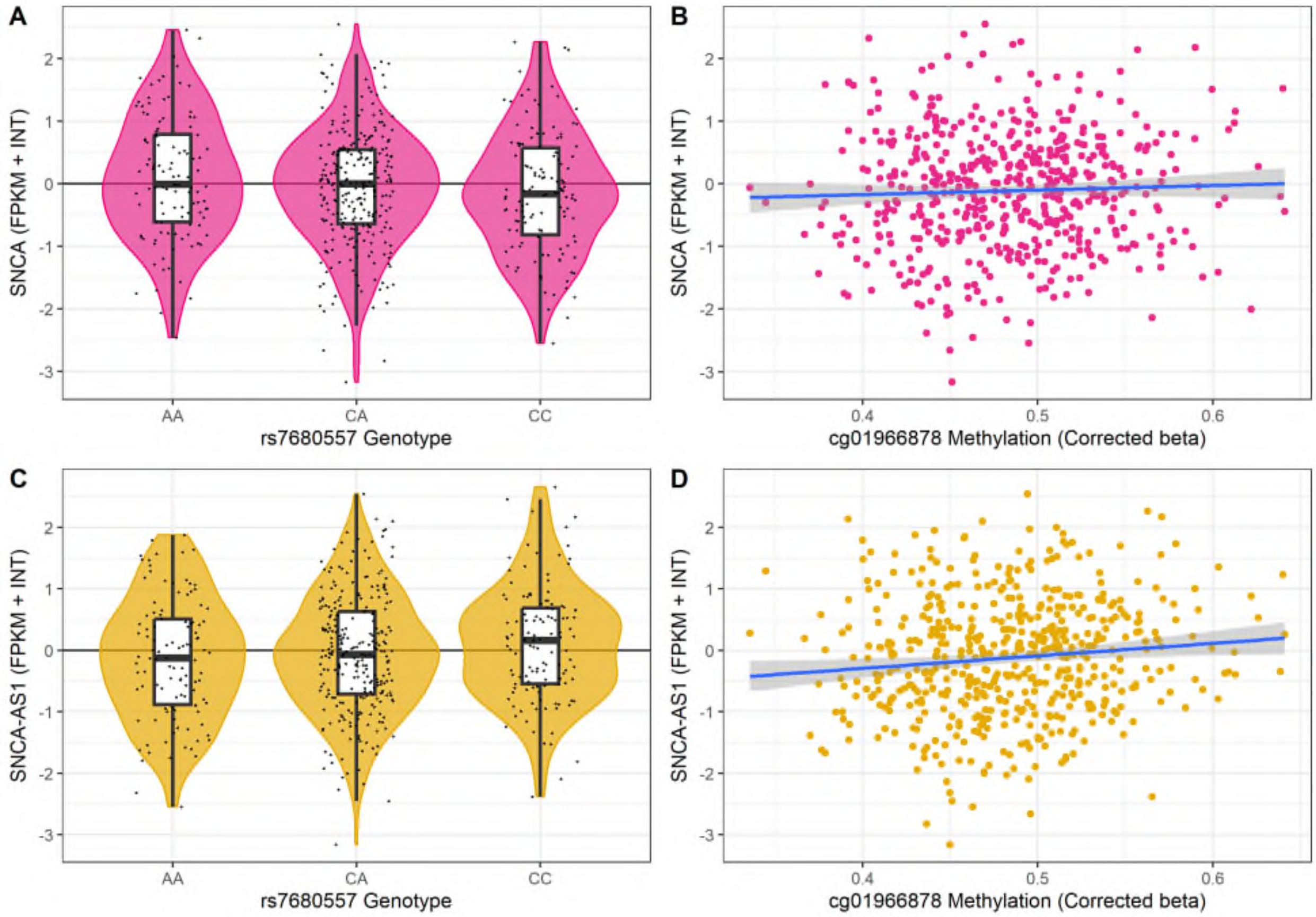


**Supplementary Fig. 5**: **Exploration of *SNCA* and *SNCA-AS1* transcripts using short read RNA-sequencing data in the ROSMAP cohort.** Shown are Fragments per Kilobase of transcript per Million mapped read (FPKM) and Inverse Variance normalized (IVN) expression against allele for the lead DLB associated SNP rs7680557 for **a** *SNCA* and **c** *SNCA-AS1*. Groups sizes per genotype for the A & C: AA = 110, CA = 268, CC = 126. Scatter plots are shown for **b** *SNCA* and **d** *SNCA-AS1* gene expression against DNA-methylation at cg019668787 (linear regression corrected beta values) within the *SNCA* gene region. The line shows the linear model-based line of best fit. 526 matched samples are displayed in plots **b** and **d**. Boxplot displays the median as a central line, the first and third quartiles as hinges and the whiskers display the minimum and maximum range (excluding outlying points greater than 1.5 times the interquartile range). Line on the scatterplot displays a linear model based line of best fit with 95% confidence interval shown as a shade around the line.

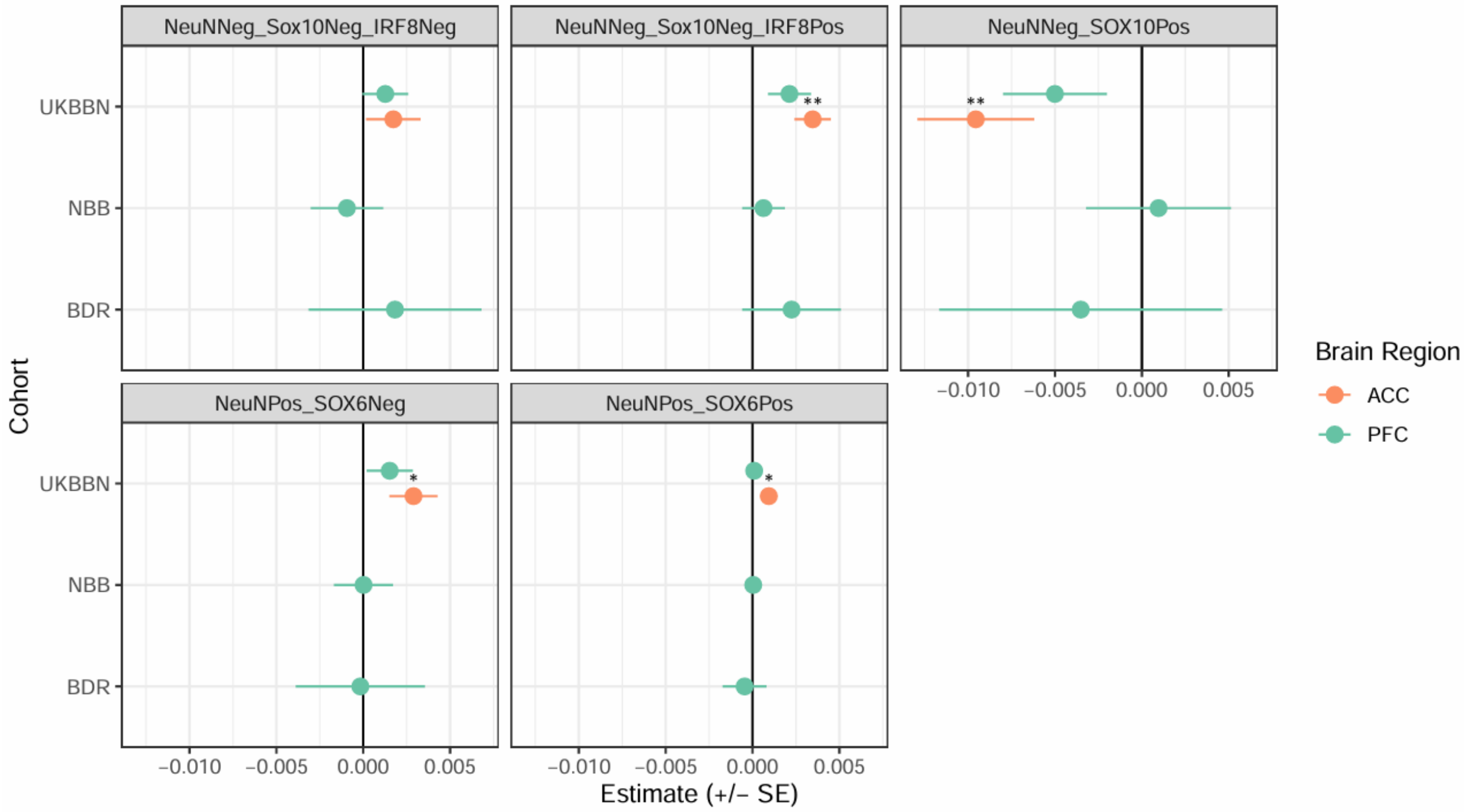


**Supplementary Fig. 6**: **Association summaries for each cohort and brain region with cell type deconvolution estimates.** Each panel shows a reference deconvolution panel from the *CETYGO* package. y-axis shows the cohort being assessed, with the color differentiating brain regions tested (ACC = Anterior Cingulate, PFC = Prefrontal Cortex). x-axis shows the association statistic for a linear model, measured in units of change per LB-Braak stage. Line represents +/- standard error around the estimate. * = nominal significance (Linear model p-value < 0.05), ** = FDR corrected significance < 0.05.
