# Supplementary material for: Interrogating DNA methylation associated with Lewy body pathology in a cross brain-region and multi-cohort study
Source: Nat Commun. 2026 Sep 15;17:10845. doi: 10.1038/s41467-026-77009-x (PMC13672336; doi:10.1038/s41467-026-77009-x)
Supplement: Supplementary file 2 [file 41467_2026_77009_MOESM2_ESM.pdf]

Description of Additional Supplementary Files

Title: Supplementary Data 1

Description: Summary of cohort demographics. Table showing the median age, interquartile range (IQR) of age, age range, median post mortem interval (PMI), IQR of PMI, PMI range, split of females (F) and males (M), and sample numbers for each stage of Lewy body (LB) and neurofibrillary tangle (NFT) Braak stage. NBB is the Netherlands Brain Bank cohort, UKBBN is the UK Brain Bank Network cohort and BDR is the Brains for Dementia Research cohort.

Title: Supplementary Data 2

Description: Summary statistics of the differentially methylated positions (DMPs) that were associated with Lewy body (LB) Braak stage in the primary full cohort LB pathology meta-analysis in cortex at a suggestive significance threshold ($p < 1 \times 10^{-5}$). Probe information is provided corresponding to chromosomal location (hg19/GRCh37 genomic annotation), Illumina (UCSC) gene annotation, closest genes with a transcription start site (TSS) upstream or downstream (from GREAT annotation). Shown for each DMP is the methylation (beta) effect size (ES), standard error (SE), corresponding unadjusted p-value (P) and false discovery rate (FDR) adjusted p-value (p_{adj}) from the cortical inverse variance fixed effects meta-analysis and the random effects meta-analysis, followed by the heterogeneity statistics (I2 and heterogeneity p-value). Also shown are the ES, SE, T-statistic (T) and unadjusted p-value from the inverse variance fixed effect model in each of the individual cohorts that were used in the cortical meta-analysis (UK brain bank network, Netherlands brain bank and Brains for Dementia Research).

Title: Supplementary Data 3

Description: Summary statistics of the 38 differentially methylated positions (DMPs) that showed a suggestive association ($p < 1 \times 10^{-5}$) with Lewy body (LB) Braak stage in the secondary pure LB pathology subset cohort meta-analysis in cortex. Probe information is provided corresponding to chromosomal location (hg19/GRCh37 genomic annotation), Illumina (UCSC) gene annotation, closest genes with a transcription start site (TSS) upstream or downstream (from GREAT annotation). Shown for each DMP is the methylation (beta)

effect size (ES), standard error (SE), corresponding unadjusted p-value (P) and false discovery rate (FDR) adjusted p-value (p_{adj}) from the cortical inverse variance fixed effects meta-analysis and the random effects meta-analysis, followed by the heterogeneity statistics (I2 and heterogeneity p-value).

Title: Supplementary Data 4

Description: Summary statistics of the 21 differentially methylated positions (DMPs) that showed a suggestive association ($p < 1 \times 10^{-5}$) with Lewy body (LB) Braak stage in the secondary Thal-controlled subset cohort meta-analysis in cortex. Probe information is provided corresponding to chromosomal location (hg19/GRCh37 genomic annotation), Illumina (UCSC) gene annotation, closest genes with a transcription start site (TSS) upstream or downstream (from GREAT annotation). Shown for each DMP is the methylation (beta) effect size (ES), standard error (SE), corresponding unadjusted p-value (P) and false discovery rate (FDR) adjusted p-value (p_{adj}) from the cortical inverse variance fixed effects meta-analysis and the random effects meta-analysis, followed by the heterogeneity statistics (I2 and heterogeneity p-value).

Title: Supplementary Data 5

Description: Summary Statistics for the 24 FDR-significant probes prioritised in the primary LB meta-analysis and their results in both subset sensitivity meta-analyses. Probe information is provided corresponding to chromosomal location (hg19/GRCh37 genomic annotation), Illumina (UCSC) gene annotation, closest genes with a transcription start site (TSS) upstream or downstream (from GREAT annotation). Shown for each DMP is the methylation (beta) effect size (ES), standard error (SE), corresponding unadjusted p-value (P) and false discovery rate (FDR) adjusted p-value (p_{adj}) from the cortical inverse variance fixed effects meta-analysis.

Title: Supplementary Data 6

Description: Summary statistics for the prefrontal cortex specific analyses. A) Differentially methylated positions (DMPs) that were associated with Lewy body (LB) Braak stage in the PFC specific meta-analysis at a suggestive significance threshold ($p < 1 \times 10^{-5}$). Probe information is provided corresponding to chromosomal location (hg19/GRCh37 genomic

annotation), Illumina (UCSC) gene annotation, closest genes with a transcription start site (TSS) upstream or downstream (from GREAT annotation). Shown for each DMP is the methylation (beta) effect size (ES), standard error (SE), corresponding unadjusted p-value (P) and false discovery rate (FDR) adjusted p-value (padj) from the cortical inverse variance fixed effects meta-analysis and the random effects meta-analysis, followed by the heterogeneity statistics (I2 and heterogeneity p-value). **B) Differentially methylated positions (DMPs) that were associated with Lewy body (LB) Braak stage in the UKBBN PFC specific EWAS at a suggestive significance threshold ($p < 1 \times 10^{-5}$)**. Probe information is provided corresponding to chromosomal location (hg19/GRCh37 genomic annotation), Illumina (UCSC) gene annotation, closest genes with a transcription start site (TSS) upstream or downstream (from GREAT annotation). Shown for each DMP is the methylation (beta) effect size (ES), standard error (SE), T-value (T) corresponding unadjusted p-value (P) and false discovery rate (FDR). **C) Differentially methylated positions (DMPs) that were associated with Lewy body (LB) Braak stage in the UKBBN ACC specific EWAS at a suggestive significance threshold ($p < 1 \times 10^{-5}$).** Probe information is provided corresponding to chromosomal location (hg19/GRCh37 genomic annotation), Illumina (UCSC) gene annotation, closest genes with a transcription start site (TSS) upstream or downstream (from GREAT annotation). Shown for each DMP is the methylation (beta) effect size (ES), standard error (SE), T-value (T) corresponding unadjusted p-value (P) and false discovery rate (FDR).

Title: Supplementary Data 7

Description: Robust rank Aggregation (RRA) prioritized genes from the primary full cohort LB pathology meta-analysis. Summary of genes prioritized by methylGSA RRA method to calculate gene ontology (GO) enrichment. Shown are the GeneID (UCSC Gene Symbol), number of probes on the EPIC array annotated to this gene (Count), Rho value from RRA test (p (rho)), Bonferroni adjusted p-value (p_{bonf}) and Lowest p-value in gene region from the fixed effect primary full cohort LB pathology meta analysis results (Min p).

Title: Supplementary Data 8

Description: Pathway analysis for the genes identified from the primary full cohort LB pathology meta-analysis for gene ontology (GO) and Kyoto Encyclopedia of Genes and

Genomes (KEGG).

Shown is the **a** GO or **b** KEGG ID, description of the pathway, the number of genes annotated to the significant DMPs in that pathway, the names of the genes annotated to the significant DMPs in the pathway, the total number of genes in the pathway, the corresponding P-value and false discovery rate (FDR)-corrected p-value (q-value).

Title: Supplementary Data 9

Description: Significant differentially methylated regions (DMRs) associated with LB pathology in the primary full cohort meta-analysis. Shown are all of the significantly associated regions that contain two or more probes from the comb-p regional analysis of the primary full cohort LB pathology meta-analysis. Shown for each DMR is the genomic region, gene annotation from UCSC, the number of significant probes in the region, Minumum p-value, Z p-value and Šidák-corrected P-value.

Title: Supplementary Data 10

Description: Overlapping methylation signatures between different neurodegenerative diseases. A) Taking the CpGs identified in our primary full cohort LB pathology meta-analysis that reached a suggestive significance threshold ($p < 1 \times 10^{-5}$, n = 67 loci) we examined whether these CpGs showed the same direction of effect in a cortical meta-analysis of Braak NFT pathology (Smith et al., 2021) and an epigenome-wide association study (EWAS) of Huntington's disease (HD) performed in the entorhinal cortex and striatum (Wheildon et al., 2026). B) Taking the Bonferroni-significant CpGs identified in the Braak-NFT cortical meta-analysis, which were present on the EPIC array in the current study (n = 208 loci**)** we examined whether these CpGs showed the same direction of effect in our primary full cohort LB pathology meta-analysis, or in the HD EWASs. The tables shows the number of loci tested (those CpGs present in the summary statistics from both studies), the number of loci showing a shared direction of effect, the Probability, p-value and false discovery rate (FDR)-adjusted p-value (p_{adj}) from a binomial sign test. For each table the two studies summary statistics compared are shown as Study 1 & Study 2. Results shown in bold were FDR significant in the binomial sign test (one sided).

Title: Supplementary Data 11

Description: Summary statistics for the primary full cohort Lewy body (LB) meta-analysis prioritized loci with respect to other neurodegenerative disease outcomes. Summary statistics table showing A) the summary statistics for the 67 CpGs prioritized in the full cohort LB meta-analysis (at suggestive significance threshold: $p < 1 \times 10^{-5}$) and their effect estimate and p-value from the LB meta-analysis, B) their effect estimate and p-value in the Braak NFT pathology cortical meta-analysis taken from Smith et al. (2021), and c and their effect estimate and p-value in the Huntington's disease (HD) epigenome-wide association study (EWAS) performed in entorhinal cortex (EC) and striatum (STR) taken from Wheildon et al. (2026). N/A denotes probes that were not analyzed in a given study.

Title: Supplementary Data 12

Description: Summary statistics for Smith et al (2021) Braak-NFT meta-analysis prioritized loci with respect to other neurodegenerative disease outcomes. Summary statistics table showing **a** the summary statistics for the 208 Bonferroni significant CpGs prioritized in the Braak NFT pathology meta-analysis (Smith et al., 2021) that were present on the EPIC array and their effect estimate and p-value in that study, **b** their effect estimate and p-value in the primary full cohort LB pathology meta-analysis performed in the current study, and **c** and their effect estimate and p-value in the Huntington's disease (HD) epigenome-wide association study (EWAS) performed in entorhinal cortex (EC) and striatum (STR) taken from Wheildon et al (2026). N/A denotes probes that were not analysed in a given study.

Title: Supplementary Data 13

Description: Methylation quantitative trait loci (mQTLs) associated with the 24 FDR significant methylated sites from the primary full cohort LB pathology meta-analysis. Shown is meta data for the methylation site tested, including the EPIC Array Methylation site ID, the manifest UCSC Gene Annotation for the site, and the chromosome (CHR) and base-pair (BP) position for that CpG site (hg19/GRCh37 genomic annotation). The QTL summary shows the number of genetic loci with an FDR significant association to that CpG site, and the start and end BP coordinates of the region of identified QTLs. For each Cpg, the topmost significant locus is summarized, with the basepair position, effect allele, distance to

the CpG site, association P-value, FDR adjusted p-value (p_{adj}) and beta association effect estimate summarized for that locus.

Title: Supplementary Data 14:

Description: Analysis of probes located in known AD, PD and DLB regions based on prior published GWAS. Brown's method was used to combine p-values (from our primary full cohort LB pathology meta-analysis) for CpGs in LD regions reported in GWAS for AD (Kunkle et al., 2019), PD (Kim et al., 2024) and DLB (Chia et al., 2021). Shown for each region are the chromosome, Region Start and Region End based of hg19 GrCh37 human genome build, the trait/study tested and corresponding citation, amd the region ID (annotated gene or nearest protein coding gene from GWAS study). N-overlap shows the number of EPIC array probes in this region that were analyzed in our primary full cohort LB pathology meta-analysis. Shown is the Brown's Scale Factor, Degrees of Freedom (DF), Uncorrected Browns aggregated p-value (P-value), and False Discovery Rate (FDR) adjusted p-value (p_{adj}) across all tests.

Title: Supplementary Data 15:

Description: Cis-methylation quantitative trait loci (mQTLs) associated with CpGs within the SNCA genetic region at FDR corrected significance. The SNP position shows the genomic location of that effect loci (hg19/GRCh37 genomic annotation) along with the effect allele at that position. Shown is summary data for the methylation site tested, including the EPIC Array Methylation site ID. The P-value of mQTL relationship, the FDR adjusted P-value (P_{adj}) and the effect Beta.

Title: Supplementary Data 16:

Description: Bayesian Colocalization analysis summary, comparing genetic risk in the *SNCA* region between GWAS nominated loci and methylation quantitative trait loci (mQTLs). N shows the number of genetic variants in the region overlapping between the tested GWAS and the mQTL summary statistics. GWAS denotes the reference trait GWAS and Methylation loci denotes the EPIC Array Methylation Site ID. Conditioned results are all sourced from the Pihlstrom 2018 analysis. Colocolization prior probabilities for each hypothesis tested is shown, with H0 = no association to either trait, H1 = Association to GWAS but not mQTL, H2

= Association to mQTL but not GWAS, H3 = Association to both GWAS & mQTL but at independent causal variants and H4 = Association to both GWAS & mQTL under the same causal variants. Evidence of colocalization, as defined by a H4 > 0.9, are bolded.

Title: Supplementary Data 17:

Description: Summary statistics of cell type deconvolution association analysis with Braak LB stage. Each row represents a specific deconvoluted cell type proportion, calculated per brain-region per cohort. Linear model summary statistics of estimate (proportion change per LB stage), standard error (SE), t-value (T), p-value (P) and false discover rate corrected p-value (FDR) are shown for each test. Tests passing FDR multiple testing correction are bolded and tests passing nominal significance are italicised and underlined.
