# Supplementary material for: Interrogating DNA methylation associated with Lewy body pathology in a cross brain-region and multi-cohort study
Source: Nat Commun. 2026 Sep 15;17:10845. doi: 10.1038/s41467-026-77009-x (PMC13672336; doi:10.1038/s41467-026-77009-x)
Supplement: Supplementary file 3 [file 41467_2026_77009_MOESM4_ESM.pdf]

nature portfolio

Corresponding author(s): Katie Lunnon

Last updated by author(s): 31st July 2026

Reporting Summary

Nature Portfolio wishes to improve the reproducibility of the work that we publish. This form provides structure for consistency and transparency in reporting. For further information on Nature Portfolio policies, see our Editorial Policies and the Editorial Policy Checklist.

Statistics

For all statistical analyses, confirm that the following items are present in the figure legend, table legend, main text, or Methods section.

n/a	Confirmed	
☐	☒	The exact sample size (*n*) for each experimental group/condition, given as a discrete number and unit of measurement
☐	☒	A statement on whether measurements were taken from distinct samples or whether the same sample was measured repeatedly
☐	☒	The statistical test(s) used AND whether they are one- or two-sided<br>*Only common tests should be described solely by name; describe more complex techniques in the Methods section.*
☐	☒	A description of all covariates tested
☐	☒	A description of any assumptions or corrections, such as tests of normality and adjustment for multiple comparisons
☐	☒	A full description of the statistical parameters including central tendency (e.g. means) or other basic estimates (e.g. regression coefficient) AND variation (e.g. standard deviation) or associated estimates of uncertainty (e.g. confidence intervals)
☐	☒	For null hypothesis testing, the test statistic (e.g. *F*, *t*, *r*) with confidence intervals, effect sizes, degrees of freedom and *P* value noted<br>*Give P values as exact values whenever suitable.*
☐	☒	For Bayesian analysis, information on the choice of priors and Markov chain Monte Carlo settings
☐	☒	For hierarchical and complex designs, identification of the appropriate level for tests and full reporting of outcomes
☐	☒	Estimates of effect sizes (e.g. Cohen's *d*, Pearson's *r*), indicating how they were calculated

Our web collection on statistics for biologists contains articles on many of the points above.

Software and code

Policy information about availability of computer code

Data collection	Data was generated on the Illumina Infinium MethylationEPIC v1.0 BeadChip, with data processing, quality control and normalization undertaken in the minfi and wateRmelon packages.Code used for data processing in this manuscript is available at https://github.com/UoE-Dementia-Genomics/LB-Meta-Analysis and includes listed versions for all packages.
Data analysis	The majority of analyses for this manuscript were performed in R (version 4.2.1) utilizing Bioconductor (version 3.16) packages. Code used in this manuscript is available at https://github.com/UoE-Dementia-Genomics/LB-Meta-Analysis and includes listed versions for all utilized packages in this analysis. DMR analysis utilizing the combined p-values package using a python conda environment (Mamba/23.1.0-4) and used the bioconda::combined-pvalues (version 0.50.6). Where applicable plink version 1.07, version 1.9 and 2 where used and are specified respectively in the text.

For manuscripts utilizing custom algorithms or software that are central to the research but not yet described in published literature, software must be made available to editors and reviewers. We strongly encourage code deposition in a community repository (e.g. GitHub). See the Nature Portfolio guidelines for submitting code & software for further information.

Data

Policy information about availability of data

All manuscripts must include a data availability statement. This statement should provide the following information, where applicable:

- Accession codes, unique identifiers, or web links for publicly available datasets
- A description of any restrictions on data availability
- For clinical datasets or third party data, please ensure that the statement adheres to our policy

Methylation data and available phenotypic data used in primary analyses for the UKBBN cohort is available on the Gene Expression Omnibus (GEO) Platform under accession number GSE341563. Data for the NBB cohort is available via GEO at accession number GSE203332. Data for the BDR cohort is available via GEO at accession number GSE197305 and additional donor data is available via Dementia Platform UK (https://portal.dementiasplatform.uk/).
Genome-wide summary statistics for meta-analyses performed in this study are available at https://github.com/UoE-Dementia-Genomics/LB-Meta-Analysis. Data has been deposited on open access repositories upon the acceptance of the manuscript, GEO Accession number GSE341563.

Research involving human participants, their data, or biological material

Policy information about studies with human participants or human data. See also policy information about sex, gender (identity/presentation), and sexual orientation and race, ethnicity and racism.

Reporting on sex and gender	In all analyses conducted, sex (here defined as a biological attribute) was included as a covariate, as the population tested included both males and females. Biological sex was confirmed based on chromosomal methylation profiles and only individuals with matching self reported sex and methylation imputed sex where included in analysis.
Reporting on race, ethnicity, or other socially relevant groupings	Ethnicity and race was inconsistently reported in available brain bank records for this analysis and was not an active inclusion or exclusion criteria in the testing conducted in this analysis.
Population characteristics	NBB Cohort:<br>Median Age: 80<br>Age IQR: 74.5–85.5<br>Age Range: 49–99<br>Sex (F/M): 149 / 173<br>Braak LB Stage Counts: (0 = 73; 1 = 6; 2 = 5; 3 = 18; 4 = 10; 5 = 81; 6 = 129)<br><br>UKBBN Cohort:<br>Median Age: 79<br>Age IQR: 74.0–84.0<br>Age Range: 47–100<br>Sex (F/M): 157 / 249<br>Braak LB Stage Counts: (0 = 90; 1 = 0; 2 = 0; 3 = 28; 4 = 44; 5 = 36; 6 = 208)<br><br>BDR Cohort:<br>Median Age: 84.5<br>Age IQR: 78-91<br>Age Range: 45–103<br>Sex (F/M): 52 / 52<br>Braak LB Stage Counts: (0 = 67; 1 = 1; 2 = 3; 3 = 9; 4 = 4; 5 = 6; 6 = 14)
Recruitment	Samples were sourced from brain banks in both the UK and the Netherlands. Thus representation of a global population is limited and potential recruitment bias may be present due to the nature of individuals likely to donate brain tissue to such centres.
Ethics oversight	Ethical approval for our study was granted from the University of Exeter Medical School Research Ethics Committee (13/02/009).

Note that full information on the approval of the study protocol must also be provided in the manuscript.

Field-specific reporting

Please select the one below that is the best fit for your research. If you are not sure, read the appropriate sections before making your selection.

[x] Life sciences [ ] Behavioural & social sciences [ ] Ecological, evolutionary & environmental sciences

For a reference copy of the document with all sections, see nature.com/documents/nr-reporting-summary-flat.pdf

Life sciences study design

All studies must disclose on these points even when the disclosure is negative.

Sample size	This study conducts primary discovery in a samples size of 1,205 samples (832 unique donors). This samples size was chosen on the basis of availability of samples and data covering cortical Lewy body pathology and utilizing the DNA methylation EPIC array for profiling.
Data exclusions	Exclusions were made for familial synucleinopathies or misdiagnosed primary symptoms (e.g., multiple system atrophy (MSA), Progressive Supranuclear Palsy (PSP) etc.), severe cerebrovascular disease or TDP-43 pathology, as well as removing cases annotated with amygdala predominant LB pathology.<br><br>Exclusions of DNA methylation data was made on the basis of standard quality control, as detailed in this papers methods section: Samples were excluded from further analysis for the following reasons: (1) they had a median methylated or unmethylated sample intensity < 1,500 or < 1,000, respectively, (2) that the bisulfite conversion efficiency was < 80%, (3) there was a mis-match between reported and predicted sex, (4) if the sample was determined to be an outlier using the outlyx function or, (5) if the samples were found to be cryptically related to one another or unrelated to its matched sample from the other brain region. Finally, using the pfilter function in the WateRmelon package samples with a detection p-value >0.05 in > 5% of probes, probes with < three beadcount in 5% of samples and probes having 1% of samples with a detection p-value > 0.05 were also removed.
Replication	To maximise discovery power, all cohorts were used in primary meta analysis and thus no replication is included here. However, the replicability of all findings is included in available summary statistics and inter-study heterogeneity statistics are included. Pathology controlled and specific sensitivity analyses have been conducted to test for specificity of primary discovery results.
Randomization	All experimental procedures were randomized in running of arrays.
Blinding	We generated DNA methylation data in the UKBBN cohort. All samples had been assigned a Brain Bank (autopsy ID), which was used for identifying the samples in the lab, with no linked information in this ID relating to sex, age or pathology. As such, although samples were not officially blinded for data generation, lab-based investigators were not able to identify samples during data processing.

Reporting for specific materials, systems and methods

We require information from authors about some types of materials, experimental systems and methods used in many studies. Here, indicate whether each material, system or method listed is relevant to your study. If you are not sure if a list item applies to your research, read the appropriate section before selecting a response.

Materials & experimental systems

n/a	Involved in the study
☒	☐ Antibodies
☒	☐ Eukaryotic cell lines
☒	☐ Palaeontology and archaeology
☒	☐ Animals and other organisms
☒	☐ Clinical data
☒	☐ Dual use research of concern
☒	☐ Plants

Methods

n/a	Involved in the study
☒	☐ ChIP-seq
☒	☐ Flow cytometry
☒	☐ MRI-based neuroimaging

Plants

Seed stocks	*Report on the source of all seed stocks or other plant material used. If applicable, state the seed stock centre and catalogue number. If plant specimens were collected from the field, describe the collection location, date and sampling procedures.*
Novel plant genotypes	*Describe the methods by which all novel plant genotypes were produced. This includes those generated by transgenic approaches, gene editing, chemical/radiation-based mutagenesis and hybridization. For transgenic lines, describe the transformation method, the number of independent lines analyzed and the generation upon which experiments were performed. For gene-edited lines, describe the editor used, the endogenous sequence targeted for editing, the targeting guide RNA sequence (if applicable) and how the editor was applied.*
Authentication	*Describe any authentication procedures for each seed stock used or novel genotype generated. Describe any experiments used to assess the effect of a mutation and, where applicable, how potential secondary effects (e.g. second site T-DNA insertions, mosiacism, off-target gene editing) were examined.*
