# Supplementary material for: Interrogating DNA methylation associated with Lewy body pathology in a cross brain-region and multi-cohort study
Source: Nat Commun. 2026 Sep 15;17:10845. doi: 10.1038/s41467-026-77009-x (PMC13672336; doi:10.1038/s41467-026-77009-x)
Supplement: Supplementary file 4 [file 41467_2026_77009_MOESM5_ESM.pdf]

nature portfolio

Peer Review File

Interrogating DNA methylation associated with Lewy body pathology in a cross brain-region and multi-cohort study

Corresponding Author: Professor Katie Lunnon

This file contains all reviewer reports in order by version, followed by all author rebuttals in order by version.

Version 0:

Reviewer comments:

Reviewer #1

(Remarks to the Author)
The manuscript by Harvey and colleagues describes the results of a study of DNA methylation of Lewy body (LB) pathology. The authors measured methylation genome-wide in a large cogort of pathological samples from the UK. The results were meta-analyzed with previously published data from two other cohorts. The main results are that the authors found a number of significant differentially methylated loci (3 or 24 dependent on the statistical threshold used) associated with LB pathology. Further, the analyses found evidence for methylation differences in the SNCA genomic region. This region is encoding alpha-synuclein, the primary pathological protein in LB diseases, highlighting that hypomethylation may be involved in disease pathogenesis.
This is a high-quality study performed by the leading research group of epigenetics in neurodegenerative disorders. The methods are sound, the interpretation of the results seems reasonable and the manuscript is well written. Figures and tables are of high quality.
In my opinion this paper is an important contribution to the literature in the field and worthy of publication in the journal. I have only one major (1) and a few very minor comments:
1. The main cohort includes DNA taken from two brain regions in most of the samples. It seems to me that methylation values for both regions are including in the primary analysis? If so, I am unsure if this is a valid strategy or will inflate the results, as the methylation values will be highly related between 2 samples from the same individual. If both regions were not included in the primary analysis, could the authors comment on the correlation between the results from the different brain regions?
2. The results are compared to other neurodegenerative disorders, including Huntington's disease. However, the samples size of the Huntington study is very small, and this limits the value of these results. This may not be fully acknowledged in the current manuscript.
3. References: Ref 1 seems to be incorrect. At least two refs are from BioRxiv. It is unclear to me if it is allowed to include refs of unpublished work.

(Remarks on code availability)

Reviewer #2

(Remarks to the Author)
In "Interrogating DNA methylation associated with Lewy body pathology in a cross brain-region and multi-cohort study" Harvey, Imm et al the authors conduct what they state is the largest EWAS for Lewy Body pathology to date.

Overall, I think this work is a solid contribution to its field.

I have a few specific comments:
1. Can anything said about the types of genomics regions the significant CpG overlap? For instance, do they enrich for predicted regulatory regions

2. The authors may want to consider editing this part of the abstract "…the most significant being located in UBASH3B and

PTAFR, and an intergenic locus" to be more transparent that those are specifically the three that reached their primary genome-wide significance threshold

3. In the "Ontological enrichment analysis…" results section, consider moving the description of the approach earlier, possibly with some edits:
"The method employed for the ontological analyses, which aggregates methylation around gene annotations to provide a set of genes to be tested for over representation analysis using robust rank aggregation (RRA)."
It was confusing reading about it after the results were already presented.

Also in this section it is said "…by performing comb-p analysis…" might want to edit for clarity and/or provide a reference

4. The discussion refers to epigenomic prediction models, but I did not see this discussed in the results

5. The authors might want to consider editing this sentence in the discussion "Notably we observe comparable cortical profiles of LB-pathology, HD-status and NFT-pathology, but minimal comparability between AD and HD profiles" to clarify how both can be the case

6. The methods section mentions "Gene ontology" but not the KEGG pathway analysis which was also used for gene set enrichments. Also since part of the approach was used for individual gene analysis separate for gene set analysis edits to this section could be made to improve clarity

7.In the abstract it is stated "…methylation quantitative trait loci in the promoter…" but in the text the claim was the "5' region of the gene" which can be broader

8. In the References for reference #1 it says "(!!! INVALID CITATION !!! 1-3)."

(Remarks on code availability)

Reviewer #3

(Remarks to the Author)
this lewy body focused DNA methylation (meta-analysis) study on cerebral cortex, with n>1200 samples to date the largest of its kind, will provide an important reference source for the field; while the total number of differentially methylated CpG after FDR correction is limited (n=24) with only 4 diff methylated 'regions', there are interesting leads such as, for example, the reported methylation changes affecting the a-synuclein encoding gene itself, including methyl-QTLs and genetic risk variants.

I have one major commment: The entire dataset is built from tissue homogenates; while there is a short paragraph in the discussion section on cell type, I feel the paper does not take advantage of current computational tools in the field to address two important questions:
a) what is the overall cell type composition of cortical neurons and glia between groups/across samples; could this be addressed to a certain degree by running cell type deconvolution analyses with their dataset?
b) usimg cell type specific mCpG reference sets, could the author narrow down the list of cell(types) that may have contributed to the altered signal at their differentially methylated positions?

also, do RNA-seq data exist for at least some of these brain specimens and if so, is there an association between gene expression and methylation?

(Remarks on code availability)

Version 1:

Reviewer comments:

Reviewer #2

(Remarks to the Author)
The authors adequately address my previous comments. I was also asked to comment on their response to Reviewer 1's comments. I think they were also adequately addressed as far as I can tell.

(Remarks on code availability)

Reviewer #3

(Remarks to the Author)
The Authors addressed sufficiently the Reviewers' comments and the paper is now much improved (with the caveat that we still have no information on gene expression changes at sites of dysregulated methylation, but ok)

(Remarks on code availability)

Open Access This Peer Review File is licensed under a Creative Commons Attribution 4.0 International License, which permits use, sharing, adaptation, distribution and reproduction in any medium or format, as long as you give appropriate credit to the original author(s) and the source, provide a link to the Creative Commons license, and indicate if changes were made.
In cases where reviewers are anonymous, credit should be given to 'Anonymous Referee' and the source.
The images or other third party material in this Peer Review File are included in the article's Creative Commons license, unless indicated otherwise in a credit line to the material. If material is not included in the article's Creative Commons license and your intended use is not permitted by statutory regulation or exceeds the permitted use, you will need to obtain permission directly from the copyright holder.
To view a copy of this license, visit https://creativecommons.org/licenses/by/4.0/

RESPONSE TO REVIEWERS

Harvey et al – Interrogating DNA methylation associated with Lewy body pathology in a cross brain-region and multi-cohort study

We thank the reviewers for the time they have taken to provide feedback on our manuscript and are grateful for the valuable feedback.

Reviewer 1:

Reviewer 1 gives a positive review on our manuscript, commenting that "...*This is a high-quality study performed by the leading research group of epigenetics in neurodegenerative disorders. The methods are sound, the interpretation of the results seems reasonable and the manuscript is well written. Figures and tables are of high quality. In my opinion this paper is an important contribution to the literature in the field and worthy of publication in the journal. ...*". They had one major comment and two minor comments, which are addressed below:

1. *"...The main cohort includes DNA taken from two brain regions in most of the samples. It seems to me that methylation values for both regions are including in the primary analysis? If so, I am unsure if this is a valid strategy or will inflate the results, as the methylation values will be highly related between 2 samples from the same individual. If both regions were not included in the primary analysis, could the authors comment on the correlation between the results from the different brain regions?..."*

   We thank the reviewer for this insightful comment regarding the regional specificity of our findings. We have now added additional analyses, where we have (1) separately analysed the prefrontal cortex and anterior cingulate cortex in the UKBBN cohort, and (2) performed a prefrontal cortex specific meta-analysis. In line with our previous sensitivity analyses, we find reduced power, but strong concordance in the primary detected effects in both the prefrontal cortex and anterior cingulate cortex specific analyses. These are now reflected in our results (pages 7-8), with additional tables (Supplementary Tables 6A-C) and figures (Supplementary Figures 1-2) having been added to the manuscript to show this data. We have also updated Figure 1 (summary of the study) to include these additional analyses

   Regarding the concern about inflation in our analyses in cohorts where more than one brain region was profiled, the model employed for the UKBBN cross cortical EWAS included a mixed-effect component to account for the non-independence of the matched samples. This is a method utilised previously for cross-cortical EWAS with matched multi-region samples, including in our own studies[1,2]. We do not see any evidence of substantial inflation driven by including these in the cross-cortical meta-analysis and in light of the strong concordance in effects identified in the updated prefrontal cortex specific analysis, we believe this cross-cortical approach is valid in this context.

2. *"...The results are compared to other neurodegenerative disorders, including Huntington's disease. However, the samples size of the Huntington study is very small,*

and this limits the value of these results. This may not be fully acknowledged in the current manuscript…"

We agree that the study size of EWAS used for summary statistic comparison will influence the capacity to identify shared or distinct signatures. We have now acknowledged this further in the discussion.

3. *"…References: Ref 1 seems to be incorrect. At least two refs are from BioRxiv. It is unclear to me if it is allowed to include refs of unpublished work…"*

   We thank the reviewer for highlighting this and apologise for the oversight and have now corrected reference 1. Our updated manuscript now only references one preprint however, that paper has already been accepted for publication, with proofs returned two months ago to the journal, so a journal citation will be available imminently, so would be included after final acceptance of the current manuscript (at the proofs stage).

<u>Reviewer 2:</u>

Reviewer 2 also provided a positive review, stating "*…I think this work is a solid contribution to its field…*". Below are our responses to their specific comments:

1. *"…Can anything said about the types of genomics regions the significant CpG overlap? For instance, do they enrich for predicted regulatory regions…"*

   We thank the reviewer for this useful suggestion. We have now performed further analysis using the knowYourCG method to explore whether the significant CpGs are enriched in specific regulatory regions. However, this analysis showed that none of the CpGs nominated from the primary meta-analysis were enriched in any specific genomic regulatory regions. We have included these results in our manuscript, under the gene set enrichment analysis section.

2. *"…The authors may want to consider editing this part of the abstract "…the most significant being located in UBASH3B and PTAFR, and an intergenic locus" to be more transparent that those are specifically the three that reached their primary genome-wide significance threshold…"*

   We have now amended the abstract to state instead "with loci located in *UBASH3B* and *PTAFR*, and an intergenic locus passing the genome wide significance threshold".

3. *"…In the "Ontological enrichment analysis…" results section, consider moving the description of the approach earlier, possibly with some edits: "The method employed for the ontological analyses, which aggregates methylation around gene annotations to provide a set of genes to be tested for over representation analysis using robust rank aggregation (RRA)." It was confusing reading about it after the results were already*

presented. Also in this section it is said "...by performing comb-p analysis..." might want to edit for clarity and/or provide a reference..."

We thank the reviewer for highlighting this. We have now moved the explanation about the RRA method to ahead of the pathway enrichment analysis. We have also now added a reference for the comb-p method.

4. *"...The discussion refers to epigenomic prediction models, but I did not see this discussed in the results..."*

   We apologise for this oversight. A previous version of our manuscript, which had been available on MedRxiv, had presented an epigenomic prediction model. However, we removed this from the final version of our manuscript submitted to Nature Communications (and the version currently on MedRxiv) both due to uncertainty of the reproducibility and generalizability of prediction models but also due to the newer *SNCA* co-localization and mQTL/eQTL findings we wished to include in the current manuscript. We have now deleted the referral to these models from the discussion.

5. *"...The authors might want to consider editing this sentence in the discussion "Notably we observe comparable cortical profiles of LB-pathology, HD-status and NFT-pathology, but minimal comparability between AD and HD profiles" to clarify how both can be the case..."*

   We thank the reviewer for highlighting this. We had meant to infer that the LB pathology epigenomic profile was comparable to the epigenetic changes seen in HD (cortex) and in AD, but that the NFT-pathology epigenomic profile was only comparable to the epigenetic changes seen in LB cortex, but not HD cortex. We have now rephrased this to improve clarity.

6. *"...The methods section mentions "Gene ontology" but not the KEGG pathway analysis which was also used for gene set enrichments. Also since part of the approach was used for individual gene analysis separate for gene set analysis edits to this section could be made to improve clarity..."*

   We thank the reviewer for highlighting this. We have now amended this section of the methods, including changing the subheading to be clearer and including details on KEGG pathway analysis.

7. *"...In the abstract it is stated "...methylation quantitative trait loci in the promoter..." but in the text the claim was the "5' region of the gene" which can be broader..."*

   We agree the current wording is vague. We have now amended the wording in the abstract to specify the 5' region rather than the promoter region.

8. *"…In the References for reference #1 it says "(!!! INVALID CITATION !!! 1-3)…"*

   We thank the reviewer for highlighting this and apologise for the oversight and have now corrected reference 1.

Reviewer 3:

Reviewer 3 also gives a positive review on our manuscript, commenting that "…*provide an important reference source for the field…*". They had one major comment and a question about availability of RNA-seq data, which are addressed below:

1. *"…The entire dataset is built from tissue homogenates; while there is a short paragraph in the discussion section on cell type, I feel the paper does not take advantage of current computational tools in the field to address two important questions: (a) what is the overall cell type composition of cortical neurons and glia between groups/across samples; could this be addressed to a certain degree by running cell type deconvolution analyses with their dataset? (b) using cell type specific mCpG reference sets, could the author narrow down the list of cell(types) that may have contributed to the altered signal at their differentially methylated positions?…"*

   We thank the reviewer for this important point and agree future work should investigate cell-type influences on methylomic signatures seen in bulk LB disease brain tissue. In reference to point (a) our study had controlled for cell type composition by including cell proportion as a co-variate in our linear models within each cohort, with these cell type proportions calculated using the CETYGO package[3]. We have also now tested for linear associations between these estimated cell type proportions and LB Braak stage separately in each cohort and in different brain regions (if available). We observed significant alterations in oligodendrocytic and microglial enriched populations in the anterior cingulate cortex (in the UKBBN cohort), but with no consistent or significant alterations observed in the prefrontal cortex in any cohort. We have now described this in the manuscript, and referred to additional new figures and tables (Supplementary Figure 6, Supplementary Table 17).

   Regarding point (b), we highlight that the current discussion point referencing Muller et al. 2025[4], is a separate paper from our group which has directly addressed this question. In that paper, using a reference dataset of 155 cortical cell-specific methylomic profiles (derived from 39 individuals), our team provided an approach to test for cell-type enrichment of EWAS findings. Taking nominally associated sites from our meta-analysis, we found a significant enrichment in microglial and oligodendrocyte enriched populations. Given these analyses already represent published work, we have not included them in this current manuscript, although we do reference this finding in the discussion.

2. *"…do RNA-seq data exist for at least some of these brain specimens and if so, is there an association between gene expression and methylation?…"*

We agree with the reviewer that it would be important to explore the association of DNA methylation and gene expression. However, at present we do not have RNA-sequencing data available in our LB cohort to allow us to explore this. However, we hope to address this in a cell type specific context, in future work building on these current findings subject to further funding.

Additional amendments:

When making amendments to our paper for this revision we noticed that we had not provided details on how we had generated the genotyping data (that was used in the mQTL analyses). We have now added a section to the methods describing genotyping data generation and analysis.

References:

1 Shireby, G., Dempster, E. L., Policicchio, S., Smith, R. G., Pishva, E., Chioza, B., . . . Mill, J. DNA methylation signatures of Alzheimer's disease neuropathology in the cortex are primarily driven by variation in non-neuronal cell-types. *Nature communications* **13**, 5620, doi:10.1038/s41467-022-33394-7 (2022).

2 Smith, R. G., Pishva, E., Shireby, G., Smith, A. R., Roubroeks, J. A. Y., Hannon, E., . . . Lunnon, K. A meta-analysis of epigenome-wide association studies in Alzheimer's disease highlights novel differentially methylated loci across cortex. *Nature communications* **12**, 3517, doi:10.1038/s41467-021-23243-4 (2021).

3 Vellame, D. S., Shireby, G., MacCalman, A., Dempster, E. L., Burrage, J., Gorrie-Stone, T., . . . Hannon, E. Uncertainty quantification of reference-based cellular deconvolution algorithms. *Epigenetics : official journal of the DNA Methylation Society* **18**, 2137659, doi:10.1080/15592294.2022.2137659 (2023).

4 Muller, J., Laroche, V. T., Imm, J., Weymouth, L., Harvey, J., Reijnders, R. A., . . . Pishva, E. A cell type enrichment analysis tool for brain DNA methylation data (CEAM). *Epigenetics : official journal of the DNA Methylation Society* **21**, 2604360, doi:10.1080/15592294.2025.2604360 (2026).
